# Development and effects of salutogenesis program for adolescents with moyamoya disease: A randomized controlled trial

**DOI:** 10.1371/journal.pone.0284015

**Published:** 2023-10-26

**Authors:** Insun Yeom, Won-oak Oh

**Affiliations:** 1 Brain Korea 21 FOUR Project, College of Nursing, Yonsei University, Seoul, Republic of Korea; 2 College of Nursing, Korea University, Seoul, Republic of Korea; National Institutes of Health, UNITED STATES

## Abstract

**Background:**

Disease-specific interventions for management and health behavior implementation are needed to improve the health and quality of life of adolescents with moyamoya disease.

**Objective:**

This study aimed to develop a program for adolescents with moyamoya disease based on the salutogenesis theory, which focuses on the process of enhancing health through successful adaptation to external stressors, and to evaluate its effectiveness.

**Methods:**

A randomized controlled trial was performed according to the CONSORT guidelines. This preliminary research and experimental treatment were conducted at a Severance Hospital ward and outpatient clinic among 48 participants randomized into the intervention (seven sessions of salutogenesis program, n = 24) or the control group (one session of one-to-one moyamoya disease education program, n = 24) from September 6, 2018 to January 4, 2019. Changes in the following study outcomes were reported: “knowledge of moyamoya disease,” “social support,” “sense of coherence,” “moyamoya disease health behavior,” “stress,” “depression,” “subjective health status,” “frequency of ischemic symptoms,” and “quality of life”.

**Results:**

The salutogenesis program improved the knowledge and social support of adolescents with illness-related problems and helped them attain healthy behaviors and stress reduction. It was confirmed to be effective in improving their quality of life.

**Conclusions:**

The salutogenesis program for adolescents with moyamoya disease effectively improved the generalized resistance resources and *sense of coherence* in adolescents with moyamoya disease.

**Trial registration:**

Korean Clinical Research Information Service registry, KCT0006869.

## Introduction

Moyamoya disease is a chronic progressive obstructive disease of the cerebral blood vessels where abnormal microscopic blood vessels are observed at the base of the brain as the end of the arterial vessels in the brain narrow gradually for no specific reason and eventually become blocked. The term “moyamoya” means “puff of smoke” in Japanese, which describes the shape of small blood vessels formed to compensate for cerebrovascular blockages [1, 2].

Moyamoya disease has two age-at-onset patterns. A study by the Korea Health Insurance Review and Assessment Service showed that the age at first moyamoya disease diagnosis is generally in the 10–19-year range (pediatric group) for 21.6% of the patients and 50–59-year range for 30.0% (adult group) [2]. Currently, the representative surgical treatment for moyamoya disease is intended to reduce symptoms and lower the risk of cerebral infarction rather than cure the conditions [3]. Therefore, it is particularly important for patients to stay healthy to prevent complications [4].

The main clinical features of moyamoya disease are slightly different among children, adolescents, and adults. Hemorrhage is common in adults, while children and adolescents have recurrent transient ischemic attacks (TIA) [5, 6]. TIA is associated with symptoms such as hemiplegia, sensory abnormalities, speech disorders, convulsions, hypergeometric brain dysfunction (such as memory, attention, performance, and cognitive disorders), and visual impairments. These symptoms can be temporary or permanent [7, 8].

Factors that trigger clinical symptoms of moyamoya disease include situations that involve hyperventilation, such as singing, blowing on musical instruments, intense crying, excessive exercise, and eating hot or spicy food [9]. These events may induce hyperventilation. Once there is hyperventilation, this leads to decreased arterial PaCO_2_ and may induce vasodilation of normal vessels and subsequent hypoperfusion in vulnerable areas via subclavian steal [2]. Symptoms of moyamoya disease can even occur in stressful situations, and the symptoms may improve immediately within few seconds to few minutes after resolving the stressful situation. However, for a long duration, the stress will need to be managed in daily life because TIAs can progress to cerebral infarctions [10]. Therefore, stress management is a particularly important health management tool for patients with moyamoya disease. Inadequate stress management can result in life-threatening events, such as cerebral infarctions and brain hemorrhages. Many patients with moyamoya disease have been reported to have increased psychological, physical, and mental burdens because of the fear of clinical complications of moyamoya disease, leading to a decline in their quality of life [11]. Therefore, health care interventions are required to minimize the factors that trigger the clinical symptoms of moyamoya disease.

## Background

Adolescents with moyamoya disease experience difficulties related to various psychological challenges and tasks during development, in addition to the clinical symptoms and experiences related to moyamoya disease treatment, all of which affect their quality of life. Therefore, interventions are needed to help adolescents with moyamoya disease fully understand the disease and improve their quality of life through effective stress management. Hence, we considered the salutogenesis theory proposed by Antonovsky as a theoretical basis for developing interventions for adolescents with moyamoya disease [12]. Salutogenesis is an open system that involves active interactions with the environment, and despite the inevitable stress and disease factors experienced in everyday life, it can help individuals overcome stress through the individual internal abilities and resources to enhance health status [13].

Accordingly, we developed and applied a “Salutogenesis Program for Adolescents with Moyamoya Disease” to improve the generalized resistance resources and sense of coherence (SOC) in adolescents with moyamoya and evaluated the effectiveness of the program.

### Aim

This study aimed to develop a salutogenesis program for adolescents with moyamoya disease and evaluate the impact of the program on the well-being of adolescents.

### Research questions and hypotheses

Research question: What is the impact of the salutogenesis program in comparison to standard care on (1) generalized resistance resources and (2) sense of coherence among adolescents with moyamoya disease?

Further, we hypothesized that compared to the control group, adolescents in the intervention condition would experience (1) stress relief, (2) gain knowledge of moyamoya disease, (3) gain social support, (4) develop a sense of coherence, (5) engage in healthy behavior, (6) experience reduction in depression and (7) frequency of ischemic symptoms, (8) observe an improve their subjective health status, and (9) notice and improvement in the quality of life.

## Methods

### Design

**Fig 1:**
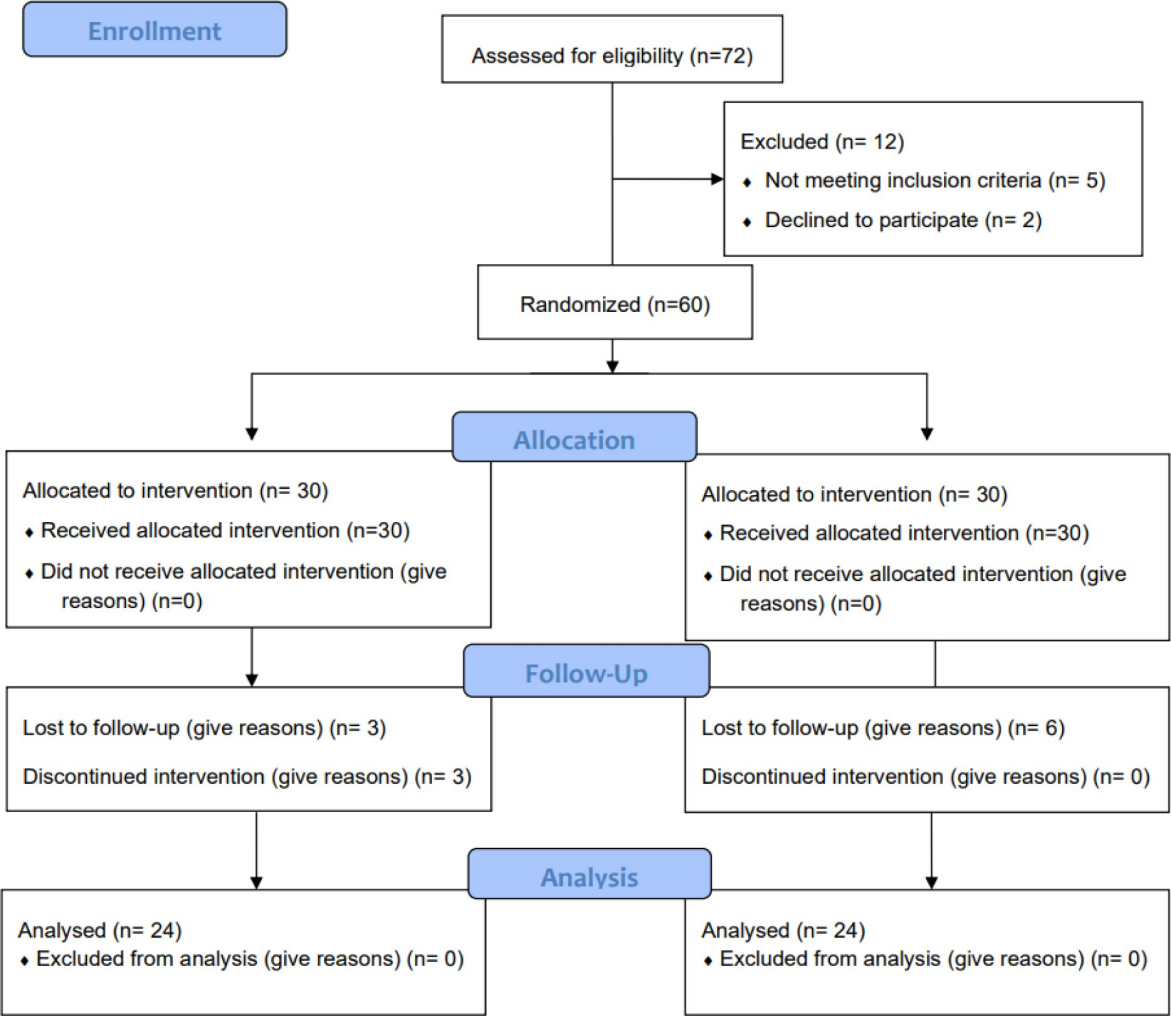
CONSORT flow chart of the study.

This study was in two phases. The first phase involved developing a salutogenesis program for adolescents with moyamoya disease. In the second phase, a parallel two-group randomized controlled trial with a prospective pretest-posttest experimental design was conducted to evaluate the effectiveness of the program. The randomized controlled trial was performed according to the CONSORT guidelines (Fig 1).

### Participants

The participants were recruited from a ward and outpatient clinic of a pediatric neurosurgery department in a hospital in Seoul, South Korea. The inclusion criteria were (a) aged 14–19 years old and (b) moyamoya disease diagnosis for more than 1 month. The exclusion criteria were a history of mental illness or difficulty in participating in the program (e.g., hearing and visual impairment).

This study is the first intervention study for adolescents with moyamoya disease, and referred to Cohen’s consumption guide for determining Sample Size [14]. The number of study participants was calculated using a two-tailed test, significance (α) level of 0.05, power of 0.8, and effect size of 0.8 using the G-power program (version 3.1.9.2; Franz Faul, Universität Kiel, Kiel, Germany) [15]. As a result, 21 participants were needed per group, but 30 participantswere selected per group, considering a dropout rate of 30%. The parallel two-group randomized controlled trial was performed from September 6, 2018, to January 4, 2019, in the ward and outpatient clinic of the pediatric neurosurgery department. The trial was registered with the *Trials Registry* Korean Clinical Research Information Service registry, KCT0006869

### Instruments

#### Stress

The stress scale [16] was used to measure the stress levels of the participants. The self-reported questionnaire consisted of 27 items, scored on a 4-point Likert scale. It included three subscales: physical stress (nine items), behavioral stress (nine items), and emotional stress (nine items). Higher scores indicated increased awareness of stress. At the time of the tool’s development, Cronbach’s alpha was 0.82; in this study, it was 0.89.

#### Knowledge of moyamoya disease

Knowledge of moyamoya disease was selected and used as an item with a content validity index of at least .80 through expert validity after the author determined the importance of knowing the disease based on evidence in the literature and clinical experience. After determining each subject’s understanding level through preliminary investigation, knowledge shortfalls were rectified. This tool was composed of four sections with 12 questions: characteristics of the disease (four questions), symptoms (two questions), treatments (two questions), and lifestyle management (four questions). One point was given for each correct answer, which means that those receiving 12 points had a high level of knowledge of moyamoya disease. In this study, the Kuder-Richardson 20 value of .81 was used to confirm the reliability of the binary variable.

#### Social support

A translated Child and Adolescent Social Support Scale (CASSS) was administered to each participant [17]. The self-reported questionnaire consisted of 48 items, scored on a 6-point Likert scale. It included four subscales: support from teachers (12 items), support from parents (12 items), support from classmates (12 items), and support from close friends (12 items). Higher scores indicated the presence of increased social support in each area. Cronbach’s alpha was .93.

#### Sense of coherence

The sense of coherence scale [13] was used to measure each subject’s SOC. The author translated the tool into Korean and reverse-translated it back to English to ensure accuracy. The scale was developed into two formats: the original measured 29 items and the shortened tool measured 13 items. Since the reliability and validity of the shortened tool were verified, we used the shortened tool for convenience.

The self-reported questionnaire consisted of 13 items, scored on a 7-point scale, resulting in a score range of 13–91. It included three sections: comprehensibility (five items), manageability (four items), and meaningfulness (four items). Higher scores indicated higher SOC. Cronbach’s alpha was .87.

#### Health behavior

The Moyamoya Health Behavior Scale (Moyamoya-HB Scale) developed by the author was used to determine the health behavior of adolescents with moyamoya disease [18]. The self-reported questionnaire consisted of 12 items, scored on a 5-point scale; therefore, the score range was 12–60. It included four sections: treatment of moyamoya disease (four items; definition of moyamoya disease, prevalence, chronic progression, and characteristics of diseases that require symptom management because cure and prevention are impossible), health promotion (four items; types of neurological symptoms of moyamoya disease and symptoms that require emergency treatment), coping (two items; medication treatment methods and surgical treatment methods), and symptoms (two items; healthy life related moyamoya disease, cerebral ischemia prevention management, stress management, prevention dehydration). Higher scores indicated better health behaviors related to moyamoya disease. At the time of developing the Moyamoya-HB Scale, Cronbach’s alpha was .86; however, it is .91 in this study.

#### Depression

We used the Korean depression scale that was corrected and supplemented by Cho Soo-cheol [19]; it was extracted from the Children’s Depression Inventory (CDI) and translated into Korean. The self-reported questionnaire consisted of 27 items: five related to emotions, seven to emotional behavioral disorders, seven to emotional interest, four to self-deprecation, and four to physiological symptoms. Higher scores indicated higher levels of depression. Cronbach’s alpha was .91.

#### Subjective health status

Subjective health status, i.e., the subjective evaluation of perceived health, was determined using a simple question scale, scored on a 5-point Likert scale, ranging from 1 point (very bad) to 5 points (very good).

#### Frequency of ischemic symptoms

The frequency of ischemic symptoms covered numbness, sensory disorder, speech disorder, and visual impairment. The daily frequency of symptoms was reported.

#### Quality of life

The quality of life scale of Kook and Varni [20] was used after translation to Korean; it was based on the Pediatric Quality of Life Inventory™ 4.0 Generic Core Scales developed by Varni, Katz, Seid, and Kurtin [21]. The self-reported questionnaire consisted of 23 items, scored on a 5-point scale. It included four subscales: physical (eight items), emotional (five items), interpersonal (five items), and school functions (five items). Higher scores indicated better quality of life. The Cronbach’s alpha value was .91.

### Development of “Salutogenesis program for adolescents with moyamoya disease”

#### Conceptual framework

The conceptual framework of this study was based on the salutogenesis theory [13] and the results of previous studies [15]. It is assumed that the ability to understand (comprehensibility), control and manage (manageability), and impart appropriate meaning (meaningfulness) can help maintain a healthy state through an improved sense of coherence [22]. Therefore, it is assumed that when a salutogenesis program is provided to adolescents with moyamoya disease, their generalized resistance resources and sense of coherence will improve.

**Fig 2:**
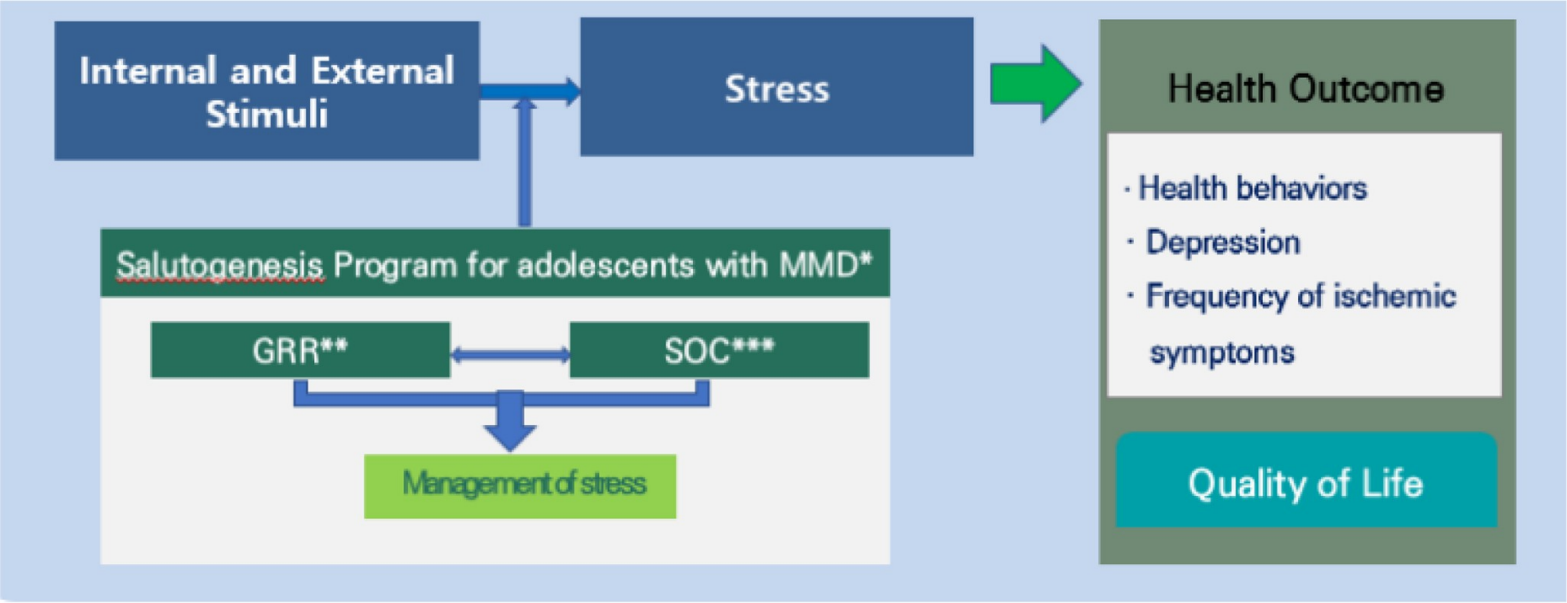
Research framework. *MMD: Moyamoya Disease, **GRR: Generalized Resistance Resources, ***SOC: Sense of Coherence.

The salutogenesis program for adolescents with moyamoya disease aims to improve their knowledge of disease, social support, and sense of coherence [23–25]. It is assumed that these elements will ultimately positively affect individuals’ quality of life by increasing healthy behavior and improving their health status [26, 27]. The participants were expected to evaluate their perception of external stressors [28], achieving their health behaviors, and increasing their health status (depression, frequency of ischemic symptoms, and subjective health status) [29]. Fig 2 shows the research framework of the study.

#### Program development process

**Fig 3:**
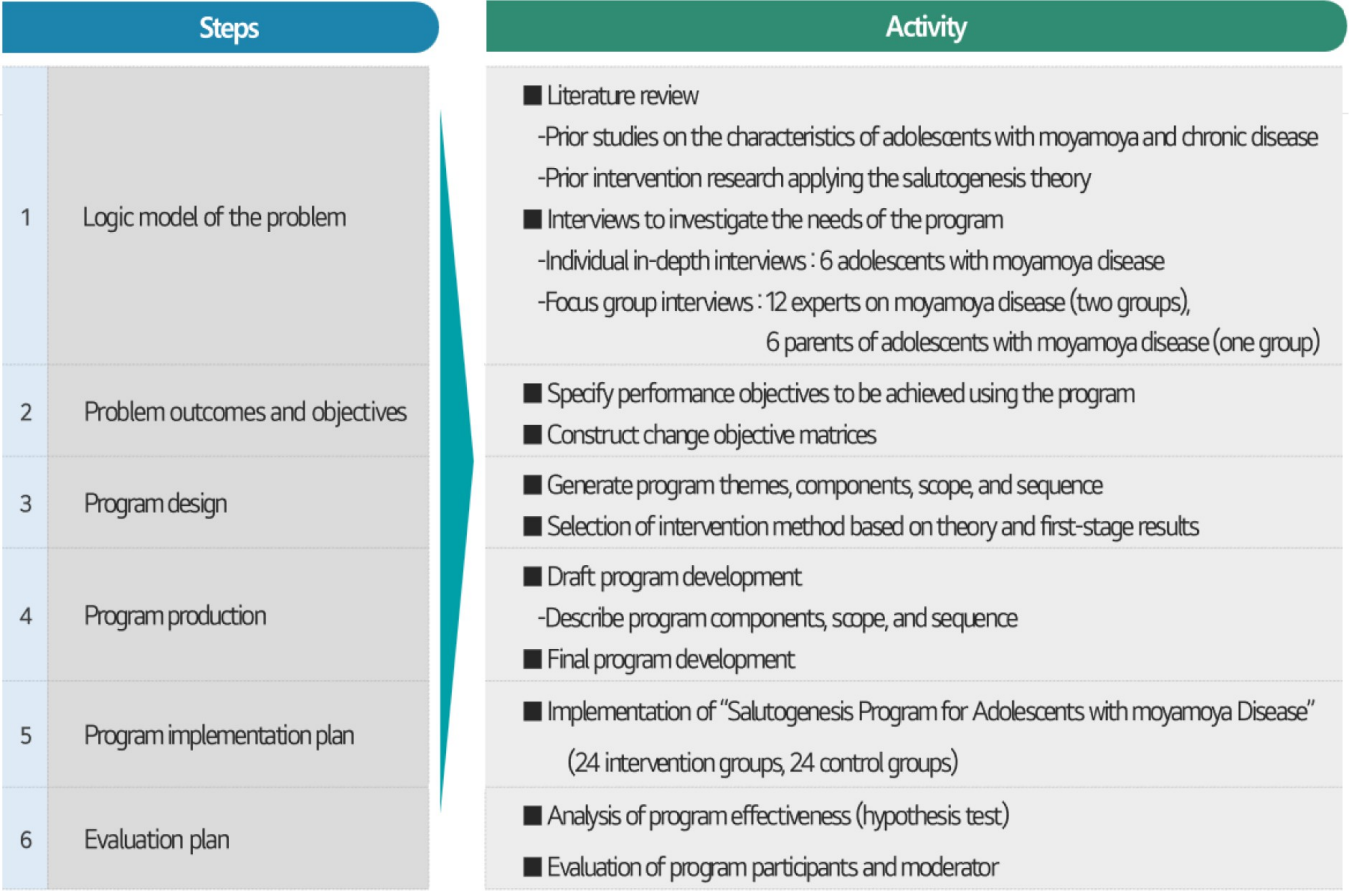
Process of program development based on the intervention mapping protocol.

The intervention mapping protocol, a widely used method for designing a systematic and evidence-based intervention program through a logical planning process, was applied to develop the salutogenesis program [30]. The intervention mapping protocol is suitable for identifying key health problems or personal and environmental factors related to health promotion and help select the most appropriate method and strategy for the identified determinants. The method consists of six steps: (1) logic model of the problem, (2) program outcomes and objectives, (3) program design, (4) program production, (5) program implementation plan, and (6) evaluation (Fig 3). The program comprised seven basic interventions for generalized resistance resources (cognitive, emotional, interpersonal relational, macro-sociocultural) and sense of coherence (comprehensibility, manageability, meaningfulness). The salutogenesis program consisted of one group education session, three group discussions, and three individual counseling sessions. The final program was revised by a group of nine experts (three nursing professors, two pediatric neurosurgeons, three neurosurgery nurse practitioners, and one psychologist with more than 10 years of experience in psychological counseling for patients with moyamoya disease). The program involved both group and individual approaches. The group approach (group education and group discussion) was designed to promote the positive effects of peer interaction [31]. The individual approach (individual counseling) reflected the unique competence of each participant based on the theme and specific content of each session and was designed for the effective operation of the program through individual feedback.

#### Evaluation of the salutogenesis program for adolescents with moyamoya disease

All participants who consented to participate in the study were assigned to intervention and control protocols through randomization. For the pre-and post-test evaluation, data were collected from patients by trained nurses.

All participants in the intervention group underwent seven sessions of the program. All programs were conducted by one neurosurgery nurse with 14 years of experience in nursing and education for moyamoya disease to maintain consistency between sessions. A surgical nurse with 3 years of experience assisted with the program.

The program sessions were conducted once weekly for 7 weeks (Table 1). Sessions 1 to 4 were conducted with small groups of 8 people who received 70 min of education or discussion every week. Session 1 was conducted under the theme of “Knowing Moyamoya Disease: Building Resistance Resources to Maintain Health”. The education session consisted of defining moyamoya disease, characteristics, risk factors, treatment, self-management, and health behavior.

**Table 1:** Health-promoting (salutogenesis) program for adolescents with moyamoya disease.

Session	Subject	Constituent factors	Content of program	Method	Time (minute)
1	Knowledge of Moyamoya Disease: Building GRR* to maintain health	GRR* (cognitive)	∙What is moyamoya disease? ∙Epidemiology and pathological findings of moyamoya disease ∙Risk factors for moyamoya disease ∙Moyamoya disease testing and diagnosis ∙Other matters related to moyamoya disease ∙Results and prognosis of indirect surgery ∙Post-surgery management ∙Basic management guidelines in daily life	Group Education	70
2	Construction of emotional and interpersonal resources for adolescents with moyamoya disease	GRR* (emotional)	∙Strengths of emotionally close relationships with other adolescents with moyamoya disease ∙Influence of positive supportive resources in the family ∙Creating close peer relationships: group interaction ∙Engaging in human relationships offer strong support	Group Discussion I	70
3	Establishment of social resources for medical professionals specializing in moyamoya disease, and establishment of resources for positive self-perception	GRR* (inter-personal relational)	∙Understand interactions with health care professionals ∙Understand the use of specialist health care support resources ∙Share available medical resources ∙Confirm and present information about the support system in their environment ∙ Identify each patient’s strengths and potential strengths	Group Discussion II	70
4	Consolidation of moyamoya disease management resource utilization	SOC** (comprehensibility, manage-ability)	∙Talk about cases that occurred in the past due to internal and external stimuli in relation to the disease ∙Predict situations or stimuli that may occur in the daily life, listen to examples from group/friends, and apply them individually. ∙Explain the reasons for the expected stimuli in the patient’s life and discuss solutions ∙Predict disease-related situations and understand how to solve them	Group Discussion III	70
5	Adolescents with moyamoya disease who can understand and overcome the stimuli of internal and external environments	SOC** (comprehensibility, manage-ability)	∙Recalling what resources were appropriately used in stressful situations in the past ∙Self-recording of available resources needed for predictable stressful situations ∙Share experiences and effects of positive emotions, find specific ways to deal with stress and learn progressive muscle relaxation	Individual Consultation I	40
6	Adolescents with moyamoya disease, understanding “special me."	SOC** SOC** (meaningfulness) & GRR* (macro-socio-cultural)	∙To understand the “precious me” concept. ∙Recall what internal and external stressful stimuli meant to the patient in the past ∙Record how predictable stressful situations pose a challenge to patient’s life and are worth the investment to focus on. ∙Discover how a patient is capable of using social resources	Individual Consultation II	40
7	Establishment of health generation in adolescents with moyamoya disease	SOC** (comprehensibility, manageability, meaningfulness)	∙ Check the self-confidence of health management after going through the program ∙ Reaffirm the meaning of health in salutogenesis ∙ Provide praise and positive feedback on an individual’s willingness to improve a sense of coherence	Individual Consultation III	50

*GRR: Generalized Resistance Resources

**SOC: Sense of Coherence

Sessions 2 to 4 were group discussions that focused on emotional support (sharing experiences on the positive impact of social support), interaction (sharing experiences with medical staff, self-help groups, etc.), and stress management (personal stress management methods, sharing experiences of problem-solving). During group discussions, the participants were asked to discuss their experiences on the selected topic. In addition, as a relaxation therapy for stress management, abdominal breathing was taught and performed together, after which, individual counseling and education were conducted in three sessions. The necessary resources for solving problems related to family, friends, opposite sex, career path, and self were provided, and a plan was formulated to promote health promotion behaviors. All the programs were conducted by the neurosurgery nurse and an assistant nurse who had been trained in this study assisted with the program.

### Data collection

The authors confirm that all ongoing and related trials for this drug/intervention are registered. A total of 60 participants and 120 parents (60 mothers and 60 fathers) provided consent to participate in the study, and the 60 participants were randomly assigned to either the intervention group (n = 30) or control (n = 30). Randomization and implementation were conducted by trained general nurses. Randomization was followed using computer-generated random numbers (http://randomizer.org).

In each group, six participants dropped out: six patients in the intervention group had withdrawn during the study, and six in the control group were excluded because they did not participate in the follow-up period (Fig 1). In the final analysis, a total of 48 participants were included.

The participants in the intervention group engaged in the salutogenesis program for 7 weeks. The control group received one-on-one education on moyamoya disease in accordance with the existing disease clinic policy. The one-on-one education program provides education on the definition, prevalence, causes, treatment methods, disease-related health behaviors, symptoms, and follow-up methods for moyamoya disease. The training took about 15 to 20 min, and after the training, a booklet containing the contents of the training was provided.

The preliminary survey was conducted for the intervention group before the program was carried out. The post-intervention survey was conducted face-to-face after all the sessions were completed. A face-to-face survey was conducted 7 weeks after the pre-test in the control group before implementing the normal one-on-one moyamoya disease education program.

### Ethical considerations

The study was approved by the institutional review board of the hospital (code number: 4-2018-0706). The researcher explained the purpose and process of the study to potential participants and written informed consent was obtained from all parents who agreed to participate. The parents were informed that they could withdraw from the study at any time with no loss of benefits, and the researcher explained that the collected data would be used for research purposes only. To ensure each participant’s anonymity and confidentiality, the collected personal information was coded and stored in an electronic file with a password, and the documented data were stored in a locked bookshelf.

### Data analysis

Data were analyzed using SPSS 20.0 for Windows. Basic information on participation was summarized using frequency, percentage, mean, and standard deviation. The homogeneity of the two groups was assessed by Fisher’s exact test, t-test, and a chi-squared test. A Shapiro–Wilk test was used to test for normal distribution of data. Paired t-tests were used to test the hypotheses. The Cronbach’s alpha coefficient and Kuder-Richardson 20 were used to test the reliability of the measurement tools.

## Results

### Demographic and clinical characteristics of adolescents with moyamoya disease

A total of 48 adolescents with moyamoya disease (intervention group, 24; control group, 24) were included in this study. The demographic and clinical characteristics of the two groups are shown in Table 2. No significant differences were found between the two groups. Additionally, the homogeneity test for pre-dependent variables did not show any statistically significant differences between the groups in all dependent variables, which confirms the homogeneity between the two groups.

**Table 2:** Demographic characteristics of adolescents with moyamoya disease in the intervention and control groups and the homogeneity of dependent variables at baseline between the two groups.

Variables		Intervention(N = 24) Mean±SD or n (%)	Control (N = 24) Mean±SD or n (%)	χ^²^ or t	*p*
**Demographic characteristics**					
Age (years)		15.25±0.69	15.47**±**0.22	-0.84	.412
School	Middle	16(66.7)	15(62.5)	0.28	.594
	High	8(33.3)	9(37.5)		
Gender	Male	13(54.2)	11(45.8)	0.44	.720
	female	11(45.8)	13(54.2)		
Religion_*_	Yes	22(91.8)	18(75.0)	3.27	.207
	No	2(8.3)	6(25.0)		
**Disease-related characteristics**					
Disease period (months)		19.76±8.63	20.33±15.55	0.26	.814
Disease stage_*_ (Suzuki stage)	2	2(8.3)	4(16.7)	0.28	.597
	3	22(91.7)	20(83.3)		
Diagnosed path_*_	clinical symptom	23(95.8)	24(100)	0.14	.932
	medical examination	1(4.2)	0(0)		
Experienced TIA _*_	Yes	23(95.8)	24(100)	0.32	.516
	No	1(4.2)	0(0)		
Surgery (EDAS) _*_	No	1(4.2)	2(8.3)	0.27	.862
	one side	8(33.3)	6(25.0)		
	both side	15(62.5)	16(66.7)		
**Homogeneity of dependent variables**					
Stress	2.11±0.56	2.02±0.46	0.30	.764
GRR	Knowledge of moyamoya disease	8.50±1.62	8.88±1.30	-1.62	.119
	Social support	4.43±0.58	4.47±0.53	-0.50	.621
SOC		61.22±10.52	63.58±9.10	-0.31	.278
Health behavior of moyamoya disease		3.24±0.56	3.54±0.53	-1.17	.212
Health status	Depression	36.78±12.37	31.78±14.94	1.12	.241
	Subjective health status	2.97±0.35	3.00±0.450	-0.29	.775
	Frequency of ischemic symptoms	0.54±0.78	0.50±0.66	0.57	.575
Quality of Life		74.76±19.27	76.25±18.97	-0.28	.749

^*^ Fisher’s exact test

TIA: Transient Ischemic Attack

EDAS: Encephaloduroarteriosynangiosis

GRR: Generalized Resistance Resources

SOC: Sense of Coherence

### Prior normality and homogeneity tests for dependent variables

The Shapiro–Wilk test to check the prior normality of the dependent variables yielded results, including stress (W = .964, p = .687; W = .917, p = .143), moyamoya disease knowledge (W = .926, p = .174; W = .968, p = .243), social support (W = .932, p = .199; W = . 961, p = .241), integration (W = .942, p = .353; W = .936, p = .273), moyamoya disease health behavior (W = .924, p = .223; W = .909, p = .107), depression (W = .936, p = .194; W = 954, p = .580), incidence of cerebral ischemia (W = .941, p = .194; W = 978, p = .693), subjective health status (W = .942, p = .354; W = .935, p = .273), quality of life (W = .954, p = .752; W = .939, p = .343), for the intervention and the control groups, respectively. Hence, normality was confirmed, and parametric analysis was used.

The result of the homogeneity test for the dependent variables of the intervention and the control groups showed that there was no statistical difference between the two groups in all variables (p>.05); hence, the homogeneity between the two groups was confirmed (Table 3).

**Table 3:** Homogeneity test for dependent variables between the groups.

Variables		Intervention group (n = 24)	Control group (n = 24)	χ^²^ or t	*p*
		M±SD	M±SD		
Stress		2.11±0.56	2.02±0.46	0.30	.764
Generalized Resistance Resources	Knowledge of moyamoya disease	8.50±1.62	8.88±1.30	-1.62	.119
	Social support	4.43±0.58	4.47±0.53	-0.50	.621
Sense of Coherence		61.22±10.52	63.58±9.10	-0.31	.278
Health behavior		3.24±0.56	3.54±0.53	-1.17	.212
Health	Depression	36.78±12.37	31.78±14.94	1.12	.241
	Frequency of ischemic symptoms	0.54±0.78	0.50±0.66	0.57	.575
	Subjective health status	2.97±0.35	3.00±0.450	-0.29	.775
Quality of life		74.76±19.27	76.25±18.97	-0.28	.749

### Effectiveness of the salutogenesis program for adolescents with moyamoya disease

Table 4 shows the pre- and post-scoring changes for each variable to test the effectiveness of the salutogenesis program. Independent and paired t-tests were performed to compare each variable score between the groups.

**Table 4:** Comparison of dependent variables between the two groups.

Variables	Pre-test	Post-test	Difference	*T* value	*p*
	Mean±SD				
Stress					
Intervention group	2.11±0.56	1.70±0.55	-0.41±0.49	3.17	.001
Control group	2.02±0.46	2.06±0.44	0.04±0.48	0.35	.791
t(*p*)	0.30(.764)	-1.69(.047)	2.73(.039)		
Knowledge of moyamoya disease					
Intervention group	8.50±1.62	10.92±0.59	2.42±1.56	7.60	<.001
Control group	8.88±1.30	10.67±0.87	1.96±1.20	7.22	<.001
t(*p*)	-1.62(.119)	1.30(.207)	1.82(.042)		
Social support					
Intervention group	4.43±0.58	4.80±0.16	0.37±0.42	9.71	< .001
Control group	4.47±0.53	4.55±0.44	0.08±0.05	1.12	.671
t(*p*)	-0.50(.621)	4.27 (< .001)	3.82(.012)		
Sense of Coherence					
Intervention group	61.22±10.52	72.34±3.91	11.12±10.26	3.71	< .001
Control group	63.58±9.10	64.78±8.81	1.00±8.92	0.41	.771
t(*p*)	-0.31(.278)	1.79(.046)	2.22(.040)		
Healthy behavior					
Intervention group	3.24±0.56	4.56±0.17	1.32±0.47	4.49	< .001
Control group	3.54±0.53	4.02±0.39	0.48±0.59	2.47	.037
t(*p*)	-1.17(.212)	1.42(.214)	2.25(.040)		
Depression					
Intervention group	36.78±12.37	34.76±10.31	-2.02±13.07	1.02	.319
Control group	31.78±14.94	30.08±15.01	-1.70±15.02	0.04	.967
t(*p*)	1.12(.241)	1.02(.311)	-0.54(.492)		
Frequency of ischemic symptoms					
Intervention group	0.54±0.78	0.50±0.59	0.04±0.79	0.44	.664
Control group	0.50±0.66	0.46±0.59	0.04±0.79	0.44	.664
t(*p*)	0.57(.575)	0.37(.714)	0.09(.982)		
Subjective health status					
Intervention group	2.97±0.35	4.31±1.11	1.33±1.79	3.08	.005
Control group	3.00±0.45	3.47±0.86	0.48±1.39	1.24	.145
t(*p*)	-0.29(.775)	2.91(.009)	2.07(.042)		
Quality-of-life					
Intervention group	74.76±19.27	82.65±18.21	7.88±19.50	3.41	.004
Control group	76.25±18.97	76.98±19.12	0.73±17.87	0.34	.711
t(*p*)	-0.28(.749)	4.12(.002)	2.41(.031)		

### Comparison of dependent variables between the two groups

Hypotheses 1–5, 8, and 9 were supported as changes in the pre- and post-scores were lower in the control group compared to the intervention group, and these changes were statistically significant. Scores were -0.41 vs 0.04(t = 2.73, p = .039), 2.42 vs 1.96(t = 1.82, p = .042), 0.37 vs 0.08(t = 3.82, p = .012), 11.12 vs 1.00 (t = 2.22, p = .040), and 1.32 vs 0.48(t = 2.25, p = .040), 1.33 vs 0.48(t = 2.07, p = .042), 7.88 vs 0.73(t = 2.41, p = .031) for hypotheses 1, 2, 3, 4, 5, 8, and 9 respectively. However, hypotheses 6 and 7 were not supported because there were no statistically significant differences in the changes between the intervention and the control groups. values were 2.02 vs 1.70(t = -0.54, p = .492) and 0.04 vs 0.04(t = 0.092, p = .982), respectively.

## Discussion

Our salutogenesis program for adolescents with moyamoya disease significantly affected the generalized resistance resources. It reduced stress and improved the sense of coherence, healthy behavior, subjective health conditions, and quality of life of adolescents with moyamoya disease.

### Stress

In this study, group discussions with a self-informational thinking induction strategy were provided to help adolescents with moyamoya disease predict their stimuli, utilize appropriate resources, and understand the value of positive stress in life. Listening to group members’ opinions effectively reduced stress in adolescents with moyamoya disease. In addition, a progressive muscle relaxation method was taught in the program, and the gradual muscle relaxation method was implemented to reduce the symptoms of physiological and psychosocial stress by inducing a transition from the arousal state to the recovery state by deactivating the sympathetic system and activating the parasympathetic system [32]. Providing a muscle relaxation music CD to be listened to at home also has positive results.

### Knowledge of moyamoya disease

The program increased the knowledge score of moyamoya disease, which is consistent with the results of studies that measured the degree of knowledge after conducting the program in adolescents [33]. In addition, the health creation program was effective in increasing the social support score. This is consistent with the findings of Estre et al. [34], who noted that social support had improved after applying a community farm experience program for general youths using the theory of health creation. Previous studies that applied a group play approach while simultaneously applying a health development theory-based integration program to different age groups significantly improved social support among peers [35, 36].

### Social support

In this study, group education and group discussion were conducted in the first four sessions and were found to have played a positive role in peer interaction and social support. In the control group, it is thought that face-to-face training by professional medical personnel played a role in providing social support to the participants. Therefore, as shown in a previous study [37], social activities and various programs should be explored to connect adolescents to the community support system.

### Sense of coherence

The program effectively improved the sense of coherence of the participants, consistent with the study results of Lövheim et al. [15], who confirmed a change in sense of coherence after applying the cognitive-behavioral intervention program for adolescents. The specific disease management and coping actions taken for the management of moyamoya disease and the motivation to talk about their thoughts to positively accomplish the developmental tasks may have enhanced the participants’ sense of coherence, consistent with the results of a previous study [38] where sense of coherence enhancement program, based on the salutogenesis theory, was applied to older patients with metabolic syndrome. In addition to group education and group discussion, an in-depth approach was adapted through three individual consultations. The intervention effectively increased the scores of healthy behaviors, consistent with Froisland and Arsand [39], who provided a web-based integration promotion program based on the theory of health creation in adolescents with type 1 diabetes.

### Healthy behavior

As a result of applying the salutogenesis program in this study, the intervention group was found to be effective in increasing the health behavior score of moyamoya disease compared to the control group. This is consistent with the research results of Froisland and Arsand [39], who provided a web-based sense of coherence enhancement program based on the theory of health creation for adolescents with type 1 diabetes. The program of Froisland and Arsand [39] allows participantsto visualize and feed back health behavior data on their own through items that can be self-evaluated by recording daily food intake, drug administration, and exercise amount along with specific education on diabetes self-management. Intervention strategies were used, and as a result, health behavior performance was improved.

In this study, the health behaviors necessary for adolescents experiencing moyamoya disease tomanage symptoms, maintain health, and cope are included. The intervention method of visualizing and delivering the data in Power Point Presentation(PPT) was applied. In addition to a booklet with the same contents as educational materials was additionally provided, but also after providing the program for each session, the participantswere provided with a task to review and reinforce the education strategy. It is interpreted as having the same effect as Froisland and Arsand [39].

### Depression

As a result of applying the salutogenesis program in this study, the effect on reducing the subject’s depression was not statistically significant. This is different from the study result of Matingwina [40], who reported that depression statistically significantly decreased as the sense of coherecnce increased after applying the intervention program for improving the integrative ability based on the theory of health generation for adolescents in general schools. In the study of Matingwina [40], the participantswere general students without disease, and the effect was analyzed after applying group activities that can improve self-identity for one semester for more than 3 months. The results are considered to be reported. Therefore, in future studies, it is thought that it is necessary to apply psychological interventions that can positively affect the emotional health status of adolescents with moyamoya disease by extending the intervention period. Future studies should also be for a longer period of time to show more significance.

### Frequency of ischemic symptoms

As a result of this study, the effect on reducing the frequency of ischemic symptom was not statistically significant. The following factors can be considered for these results of this study.

First, although the program of this study was effective in inducing positive changes in the knowledge and health behaviors of adolescents with moyamoya disease, the lack of improvement in the frequency of cerebral ischemia indicates that the measurement index did not sensitively reflect neurological symptoms. That is, only the frequency of ischemic symptom was measured, and it is considered that clinical symptom indicators such as the intensity and duration of neurological abnormalities were not sufficiently reflected. Therefore, in future studies, it is suggested to apply various outcome scales that can be measured by sufficiently reflecting the clinical characteristics of moyamoya disease.

Second, both the intervention group and the control group participating in this study corresponded to the relatively stable group when judging the severity of moyamoya disease. That is, not only the study participant but also the participant’s parent fully understood the purpose of this study and agreed to participate. The participantswere in a relatively stable stage who were receiving regular check-ups afterward or who were currently undergoing regular check-ups to check the progression of the disease without a surgical plan. Therefore, in future research, it is suggested to conduct a study design and intervention that considers the severity or stage of moyamoya disease (Suzuki stage) as a block factor [1].

### Subjective health status

In addition, the intervention was found to be effective in improving the scores on subjective health status. This is consistent with a study in which the subjective health satisfaction score shows a statistically significant increase after 10 months of exercise therapy, based on the salutogenesis theory, in an older group [41]. However, in the case of previous studies, unlike this study, a study that provided a long intervention of 10 months may have additionally affected fidelity. The program of Kohut et al. [41] provided details on specific exercise and stress management methods to improve the health and immunity among older patients and provided self-care and strategies for disease prevention; their intervention had positive effects.

### Quality of life

Finally, the program had a positive effect on the quality of life of the participants. Quality of life includes physical, mental, and social well-being and subjective satisfaction that individuals perceive as they live [21]. Therefore, when carrying out the program, overall disease-related knowledge, personal health awareness, positive values and attitudes toward life, and the ability to recognize and utilize available resources should be considered to improve users’ quality of life.

Moyamoya disease, a cerebrovascular disorder, is the biggest cause of stroke in children and adolescents, and as a secondary problem, it is a disease that burdens the cardiovascular system, so early management is very important. Moyamoya disease is a rare ailment, and as its incidence has increased recently, there have been many inquiries about its treatment and nursing care. It is also part of the group of diseases that need additional clinical exploration for the benefit of patients. Further, recently the number of lectures and conferences related to moyamoya disease has gone up in North America and Europe. With the recent deployment of numerous conferences and studies on moyamoya disease in North American and Europe, this study is of great significance as a basis for mid-level and interventional research to improve the health of adolescents with moyamoya disease.

### Limitations

This study has several limitations. First, the participants self-reported their subjective responses in the questionnaires. Given that there may be differences in the consistency of responses, this method of investigation is not without bias. Second, since the intervention focused on disease education and resource management to effectively manage the clinical symptoms experienced by adolescents with moyamoya disease, it did not affect reducing the depression levels of the participants. Third, the duration of the study was only 4 months. This may affect the fidelity of intervention research in the enhancement of intervention skills. Fourth, when obtaining the sample size in this study, the reliability of the hypothesis test results is moderate in that a relatively medium power value was used as a study conducted on adolescents with rare intractable diseases. Finally, the generalization of the effectiveness of the program is limited because the study involved adolescents with moyamoya disease at a single hospital in Seoul, Korea.

## Conclusion

This study aimed to develop a salutogenesis program for adolescents with moyamoya disease and evaluate its effects. The salutogenesis program was confirmed to be effective in stress relief, gain knowledge of moyamoya disease, gain social support, develop a sense of coherence, engage in healthy behavior, observe an improve their subjective health status, and notice and improvement in the quality of life. However, it was confirmed that depression, a variable of psychological health status, and frequency of ischemic symptoms, a variable of physical health, were not effective in this study. This is considered to be a limitation that this program was difficult to improve the clinical symptoms and emotional state of adolescent patients with moyamoya disease.

However, in spite of this, in terms of subjective health status and quality of life, this program is considered beneficial and necessary for adolescents with moyamoya disease. Therefore, by applying it to future research or hospital practice, it reduces stress by increasing disease-related knowledge, social support, and csense of coherence among adolescents with moyamoya disease.

## Supporting information

S1 File Raw data. Dataset.
(XLSX)

S2 File Protocol for study. (DOC)

S3 File (DOC)

S1 Checklist CONSORT 2010 checklist of information to include when reporting a randomised trial*. (DOCX)
