# Supplementary material for: Development and effects of salutogenesis program for adolescents with moyamoya disease: A randomized controlled trial
Source: PLoS One. 2023 Oct 26;18(10):e0284015. doi: 10.1371/journal.pone.0284015 (PMC10602295; doi:10.1371/journal.pone.0284015)

**Protocol for Study**

**Title**

Development and effects of Salutogenesis Program for Adolescents with Moyamoya Disease: a Randomized Control Trial.

**1. Scientific basis for research**

Moyamoya disease is a chronic progressive obstructive disease of the cerebral blood vessels where abnormal microscopic blood vessels are observed at the base of the brain as the end of the arterial vessels in the brain gradually narrow for no specific reason and eventually become blocked. The term “moyamoya” means “puff of smoke” in Japanese, which describes the shape of small blood vessels formed to compensate for cerebrovascular blockages. Moyamoya disease has two age-at-onset patterns, and data analyzed by the Korea Health Insurance Review and Assessment Service show that the age of first moyamoya disease diagnosis is in the 10- to 19-year-old range (adolescent) for 21.6% of patients, and 30.0% are aged 50-59 years (late adulthood). Currently, the representative surgical treatment of moyamoya disease is intended to reduce symptoms and lower the risk of cerebral infarction rather than focusing on a cure. Therefore, it is particularly important for patients to stay healthy to prevent complications.

The main clinical symptoms of moyamoya disease are slightly different in children, adolescents, and adults. Hemorrhage is common in adults, while children and adolescents have recurrent transient ischemic attacks [TIA]. TIA have symptoms such as hemiplegia, sensory abnormalities, speech disorders, convulsions, hypergeometric brain dysfunction (such as memory, attention, performance, and cognitive disorders), and visual impairments. These symptoms can appear temporarily or permanently.

Factors that trigger TIA include situations that involve hyperventilation, such as singing, blowing on musical instruments, intense crying, excessive exercise, and eating hot or spicy foods. TIA can even occur in stressful situations, and symptoms may improve immediately within few seconds to few minutes. However, if the duration is longer, stress may need to be managed in daily life because TIA can progress to cerebral infarctions. Therefore, stress management is a particularly more important health management tool in patients with moyamoya disease than in normal people. If stress management is not properly performed, life-threatening events, such as TIA, cerebral infarctions, and brain hemorrhages, can occur. Many patients with moyamoya disease have been reported to have increased psychological, physical, and mental burdens because of fear of TIA, which leads to a decline in their quality of life. Therefore, health care is required to minimize the factors that cause clinical symptoms of moyamoya disease.

Adolescents with moyamoya disease experience difficulties related to various psychological challenges and tasks experienced during their adolescent development in addition to clinical symptoms and experiences related to the treatment of moyamoya disease, which affects their quality of life. Therefore, intervention is needed to help adolescents with moyamoya disease fully understand the disease and improve their quality of life through effective stress management. Consequently, we consider the salutogenesis theory proposed by Antonovsky as a theoretical basis for the development of intervention for adolescents with moyamoya disease. Salutogenesis is an open system that actively interacts with the environment, and despite the inevitable stress and disease factors experienced in everyday life, it overcomes stress through the individual’s internal abilities and resources to reach health. Interventions for adolescents with moyamoya disease require the application of the salutogenesis theory so that they can overcome the stress of the disease and enhance their ability to improve their health rather than attempting to eliminate moyamoya disease.

2. Objectives

The purpose of this study is to develop a health generation program for adolescents with moyamoya disease and to test its effectiveness.

1) Develop a salutogenic program to improve the health of adolescents with moyamoya disease.

2) The effectiveness of the Salutogenic program for improving the health of adolescents with moyamoya disease is verified.

3. Methods

3.1. Intervention Group

A total of 7 sessions (7 weeks) Salutogenesis program was applied to the intervention group once a week. The specific details are as follows

3.1.1. Intervention Program Protocol

“Salutogenesis program for adolescents with moyamoya disease”

1) Session 1: Group training

Subject: Knowledge of Moyamoya Disease: Building Resistance Resources to Maintain Health

Activity time: 70 minutes

Materials to prepare: Beam screen, individual workbook

Progress: Researcher

2) Session 2: Group Discussion

Subject: Construction of emotional and interpersonal resources for adolescents with moyamoya disease

Activity time: 60 minutes

Materials to prepare: Beam screen, individual workbook

Progress: Researcher

3) Session 3: Group discussion

Topic: Building social resources for medical professionals specializing in moyamoya disease, building positive perception resources about me

Activity time: 70 minutes

Materials to prepare: Beam screen, individual workbook

Progress: Researcher

4) Session 4: Group discussion

Subject: Consolidation of Moyamoya Disease Management Resource Utilization

Activity time: 60 minutes

Materials to prepare: Beam screen, individual workbook

Progress: Researcher

5) Session 5: Individual counseling

Topic: Developing the ability to discern and interpret things related to me

Activity time: 40 minutes

Materials to prepare: individual assignment notes, notebooks

Progress: Researcher

6) Session 6: Individual counseling)

Subject: Understanding Moyamoya Disease and the Special Me in My Adolescence

Activity time: 40 minutes

What to bring: Individual workbooks, questionnaires

Progress: Researcher

7) Session 7: Individual counseling

Subject: Pursue a life of health promotion

Activity time: 50 minutes

What to bring: Individual workbooks

Progress: Researcher

3.1.2. The intervention group consisted of a total of 24 people, and the experiment was conducted on subjects between the ages of 13 and 18.

.

3.2. Control Group

Using the moyamoya disease brochure, the researcher directly conducted a 10-minute one-on-one disease education once.

3.3. Instrument

1. Stress
2. Social support
3. Sense of Coherence
4. Health behavior
5. Subjective health status
6. Frequency of ischemic symptoms

7) Quality of life

Experimental group: before the intervention, after the intervention (7 weeks later) Control group: before one-on-one education and 7 weeks after education

4. Name and address of the research institute

Severance Children's Hospital, 50-1 Yonsei-ro, Seodaemun-gu, Seoul

5. Research support institutions

None.

6. Research period

Estimated duration of study (~ 12 months from the date of IRB approval)

8. Study subjects

The subjects of this study were those who were diagnosed with moyamoya disease, who visited the moyamoya disease clinic at Severance Hospital in Seoul or were hospitalized, and those who understood the purpose of this study and signed and consented to the purpose of this study, and those who read and responded to the questionnaire. For those who can communicate and can communicate, the specific selection and exclusion criteria for steps 1 and 2 are as follows.

Level 1

The specific selection criteria for the subjects of this study are as follows.

1) Adolescents between the ages of 13 and 18 and their parents

2) Those who have been diagnosed with moyamoya disease for more than 1 month

The specific exclusion criteria for the subjects of this study are as follows.

Those who have a history of mental illness in medical records or who have a disease that interferes with the quality of program development (eg, hearing impairment, visual impairment, etc.)

Step 2

The specific selection criteria for the subjects of this study are as follows.

1) Those who have been diagnosed with moyamoya disease for more than 1 month

2) Those who can participate in the second stage research for program verification

The specific exclusion criteria for the subjects of this study are as follows.

Persons with a history of mental illness in the medical record or a disease that interferes with the program (eg, hearing impairment, visual impairment, etc.)

Assignment of subjects to the experimental group and control group will be randomized using a random number generator (http://randomizer.org) in the order of participation in the study to control for confounding variables between groups.

Random assignment uses a computer program to generate random numbers as many as the total number of subjects, then divides them into 1 and 2 based on the median value.

Assessed for eligibility (n= )

Excluded (n= )

Allocated by Randomization (n=60)

**Allocation**

Salutogenesis program care

Intervention Group (n=30)

Conventional care

Control Group (n=30)

Dropouts (n= )

Lost follow-up (n= )

Completed Study Protocol

Analysis (n= )

Control group

Analysis (n= )

Intervention group

**<Figure 1>** > CONSORT diagram

** Protection measures when recruiting vulnerable subjects

1. Adolescents with moyamoya disease are minors with cerebrovascular disease and, in the case of subjects who can communicate, explain this study in a natural environment in easy language and ask for consent.

2. Provide sufficient explanation to as many parents as possible and obtain consent for the study. If parents are not present, consent is obtained from a legal guardian.

3. Consent may be withdrawn at any time according to the will of the research participant, and there will be no disadvantages regarding treatment.

9. Estimated number of study subjects and calculation basis

In the first stage, in-depth interviews were conducted with 6 adolescents with moyamoya disease and 6 parents.

G*power 3.1.9.2 was used to calculate the number of study subjects in step 2, and the effect size of the study was reported in the study results of an intervention program applied to improve integrative power among adolescents by Perminas & Dovile (2011) among previous studies. Integration scores were used. In this study, the effect size (d) calculated using the results of the experimental group (M=45.6, SD=2.8) and the control group (M=43.3, SD=3.4) was 0.74, the significance level α= .05, and the power (1 As a result of calculating -β)=.80, the number of subjects in each group was 24, respectively. Considering the dropout rate of 25%, a total of 30 people in the experimental and control groups will be recruited, and a total of 60 people will be recruited.

The research staff will directly obtain the consent and recruit them.

10. Recruitment of study subjects

Severance Hospital Pediatric Neurosurgery or Neurosurgery for outpatient or hospitalized patients with moyamoya disease, the need for research will be explained and written consent will be given to subjects and guardians between the ages of 13 and 18. Adolescents are a vulnerable group, and they plan to participate in the study only when the subject and their guardians have consented.

11. Consent of study subjects and continuous participation in research participation

In order to obtain the written consent of the research subject, the researcher will directly receive the consent form after explaining the purpose and method of the research. As in #8, the recruitment of vulnerable subjects will be conducted according to the protection method. Also, during the study period, the person in charge of the research will confirm the continued participation before participating in the study.

12. Specific research method

This study is to be composed of a study to develop a moyamoya disease health generation program for health promotion of adolescents with moyamoya disease (step 1) and an experimental study to test the effect of the developed moyamoya disease health generation program (phase 2). Scheduled <Figure 2>.

In order to develop the program in detail, we plan to refer to the program development model of Kim Chang-dae et al. (2011), which applied the planning stage, the program composition stage, the preliminary research stage, and the program implementation and evaluation stage.

The planning stage is the stage of establishing the program goals through literature review and a survey on the needs of potential program beneficiaries, that is, youths with moyamoya disease. In this study, the target of the program, youths with moyamoya disease, focus on their potential resources rather than focusing on their problems, and aim to recognize and improve their current resistance resources. In addition, it is planned to conduct a review of related literature in order to improve the integration ability to systematize one's life and manage resources. In addition, in-depth interviews and focus group interviews with adolescents, parents, and clinical experts will be conducted.

The composition stage is the stage in which each element is organized by deriving the program elements, composition content, operation method, etc. to achieve the goal based on the results obtained in the planning stage, and composing the main content of the program that conforms to each principle. In this study, based on the literature review at the planning stage and the results of focus group interviews with clinical experts, the composition and contents of a program to enhance general resistance resources and individual integration while considering the characteristics of adolescence with moyamoya disease will be organized.

Lastly, in the program implementation and evaluation stage, the draft program obtained in the composition stage is run on a small number of experimental subjects to measure the effectiveness of the program, and the participants receive evaluative feedback. Through this, it can be said that it is the stage to improve the degree of completion of the program for generalization of the program by modifying and supplementing the activities, contents, and strategies of the program. After that, the final program will be developed after revision and supplementation through expert supervision and advice.

13. Efficacy Evaluation Criteria and Methods

The method of confirming the effect of the study was that, among the observation items, disease-related knowledge, perceived social support, cohesion, adolescent stress, health behavior, subjective health status, and quality of life of adolescents with moyamoya disease would increase through program application, whereas depression, the incidence of ischemia is expected to decrease.

14. Data Analysis and Statistical Methods

The collected data will be analyzed using SPSS WIN(20.0). The detailed method is as follows.

(1) The general characteristics of the experimental group and control group, as well as the distribution characteristics of all research variables, will be calculated with descriptive statistics such as real number, percentage, mean, and standard deviation.

(2) Shapiro-Wilk will be used for normality tests for general characteristics and dependent variables.

(3) The general characteristics and prior homogeneity verification of the experimental group and control group will be analyzed using X²-test and t-test.

(4) Hypothesis testing will be analyzed using t-test and paired t-test.

15. Criteria for Suspension and Dropout

When the target subjects of the study are recruited, data collection will end and the study will be stopped through the study analysis method. The criteria for early termination is that if it is difficult to recruit study subjects, the study will be stopped early. In addition, if the subject has a health condition that makes it difficult to continue participating in the study or a situation that affects the progress of the study occurs, the study will be stopped.

Further specific examples are as follows.

1) In case the subject withdraws consent to participate in the study (if the subject requests to stop/withdraw from the study, the study can be stopped/withdrawn at any time)

2) When it is judged that participation in the research is difficult due to the nature of the disease, such as worsening of symptoms, or due to a sudden accident.

Reference

# [**Anna, Z.**](https://www.ncbi.nlm.nih.gov/pubmed/?term=Zashikhina A%5BAuthor%5D&cauthor=true&cauthor_uid=24460738) and [**Bruno, H.**](https://www.ncbi.nlm.nih.gov/pubmed/?term=Hagglof B%5BAuthor%5D&cauthor=true&cauthor_uid=24460738) (2014). Health-related quality of life in adolescents with chronic physical illness in northern Russia: a cross-sectional study*.* [***Health Qual Life Outcomes***](https://www.ncbi.nlm.nih.gov/pmc/articles/PMC3905674/). 12(12).

1. Antonovsky, A. (1979). Health, Stress and Coping. *San Francisco*: Jossey-Bass*.*
2. Antonovsky, A. (1987). Unraveling the mystery of health: How people manage stress and stay well. *San Francisco*: Jossey-Bass.
3. Antonovsky, A. (1993). The salutogenic approach to aging. *Lecture held in Berkeley*, 2(21).
4. Antonovsky, A. (1996). The salutogenic model as a theory to guide health promotion*.. Health Promotion International*, 11(1), 11-18.
5. Aldwin, C. M., & Revension, T. A. (1987). Does coping heip? A reetamination of the relation between coping and mental health. *Journal of Personality and Social Psychology*, 53, 337-348.
6. Apers, S., Moons, P., Goossens, E., Luyckx, K., Gewillig, M., Bogaerts, K. (2013). Sense of coherence and perceived physical health explain the better quality of life in adolescents with congenital heart disease. European Journal of Cardiovascular Nursing, 12(5), 475–483.
7. Ayo, Y. O., Reddy, P. S., & Van, B. W. (2009). Longitudinal association of adolescents’ sense of coherence with tooth‐brushing using an integrated behavior change model. *Community Dentistry and Oral Epidemiology*, 37(1), 68–77.
8. Bang, O. Y., Fujimura, M., & Kim, S. K. (2016). The Pathophysiology of Moyamoya Disease. *An Update. Journal of Stroke*, 18(1), 12-20.
9. Barlow, J. H. & Ellard, D. R. (2006). The psychosocial well-being of children with chronic disease, their parents and siblings: An overview of the research evidence base. *Child: Care, Health and Development*, 32(1), 19-31.
10. Baker L.K. & Denyes M.J. (2008) Predictors of self-care in adolescents with cystic fibrosis: a test of Orem‘s theories of self-care and self-care deficit. Journal of Pediatric Nursing 23 (1), 37–48.
11. Bao, X. Y., Duan, L., Yang W. Z., Li, D. S., Sun, W. J., Zhang, Z. S., Zong, R., Han, C. (2015) Clinical features, surgical treatment, and long term outcome in pediatric patients with moyamoya disease in China. *Cerebrovasc Dis,* 39(2), 75–81.
12. Beck, A. T. (1972). Depression: Causes and Treatment*. Philadelphia*: University of Pennsylvania Press.
13. Bersano, A., Guey, S., Bedini, G., Nava, S., Hervé, D., Vajkoczy, P., Klijn, C. (2016). Research Progresses in Understanding the Pathophysiology of Moyamoya Disease. *Cerebrovascular Diseases*, 41(34), 105-118.
14. Bronikowski, M. & Bronikowska, M. (2009). Salutogenesis as a framework for improving health resources of adolescent boys. Scandinavian Journal of Public Health, 37*(*5), 525–531.
15. Bengtsson, T. A., & Hansson, L. (2001). The validity of Antonovsky’s sense of coherence measure in a sample of schizophrenic patients living in the community. *Journal of* *Advanced Nursing*, *33*(4), 432-438.
16. Braun, L. O. (2014). Coping resources and stress reactions among three cultural groups one year after a natural disaster. *Clinical Social Work Journal*, 42, 366–374.
17. Braun, L. O., Sagy, S., & Roth, G. (2010a). Coping strategies among adolescents: Israeli Jews and Arabs facing missile attacks. Anxiety Stress and Coping, 23(1), 35–51.
18. Blom, E. C. H., Serlachius, E., Larsson, J. O., Theorell, T., & Ingvar, M. (2010). Research low sense of coherence (SOC) is a mirror of general anxiety and persistent depressive symptoms in adolescent girls-a cross-sectional study of a clinical and a non-clinical cohort*. Health and Quality of Life Outcomes*, 8, 58.
19. Choi, B. Y., Kim, D. H., Chung, K. M., Park, M. J., & Lee, E. B. (2010). Psychosocial characteristics of girls with Turner syndrome and age-matched healthy control. *Korean Psychological Association Woman,* 15(3), 489-507.
20. Cho, S.C. and Lee, Y.S. 1990. Development of the Korean form of the Kovacs’ Childeren’s Depression Inventory. *Journal of Korean Neuropsychiatric Association,* 29(4): 943- 956.
21. Chmlin, S. L. & Chren, M. M. (2010). Quality of life outcomes and measurement in childhood atopic dermatitis. *Immunology and Allergy Clinics of North America*, 30(3), 281-288.
22. Delgado, C. (2007). Sense of coherence, spirituality, stress and quality of life in chronic illness. *Journal of Nursing Scholarship,* *39*(3), 229-234.
23. Dubow,E. F.,& Tisak,J.(1989).Therelation between stressfullifeevents and adjustmentin elementary schoolchildren:Theroleof social support and social problem-solving skills. *Child* *Development, 60,* 1412-1423.
24. Eriksson, M., & Lindström, B. (2005). Validity of Antonovsky’s sense of coherence scale: a systematic review. *Journal of* *Epidemiology and Community Health, 59*(6), 460-466.
25. Fujimura, M., Sonobe, S., Nishijima, Y., Niizuma, K., Sakata, H., Kure, S. (2014). Genetics and Biomarkers of Moyamoya Disease: Significance of RNF213 as a Susceptibility Gene, [*J Stroke*.](https://www.ncbi.nlm.nih.gov/pubmed/24949311) 16(2), 65-72.
26. Fok, S. K., Chair, S. Y., & Lopez, V. (2005). Sense of coherence, coping and quality of life following a critical illness. *Journal of Advanced Nursing, 49*(2), 173-181.
27. Garcı´a, M. I., Moreno, C., & Braun, L. O. (2013). Neighbourhood perceptions and sense of coherence in adolescence. The Journal of Primary Prevention, 34(5), 371–379.
28. .Garcı´a, M I., Moreno, C., & Jime´nez, I. A. (2013). Understanding the joint effects of family and other developmental contexts on the sense of coherence (SOC): A person-focused analysis using the classification tree. *Journal of Adolescence,* 36(5), 913–923.
29. Garcı´a, M. I., Rivera, F., & Moreno, C. (2013). School context and health in adolescence: The role of sense of coherence. *Scandinavian Journal of Psychology*, 54(3), 243–249.
30. Garnefski, N., Kraaij, V., & Spinhoven, P. (2001). Negative life events, cognitive emotion regulation and emotional problems. *Personality and Individual Differences,* 30(8), 1311–1327.
31. Gauffin, H., Landtblom, A. M., & Ra¨ty, L. (2010). Self-esteem and sense of coherence in young people with uncomplicated epilepsy: A 5-year follow-up. Epilepsy & Behavior, 17(4), 520–524.
32. Geckova, A. M., Tavel, P., van, D. J., Abel, T., & Reijneveld, S. (2010). Factors associated with educational aspirations among adolescents: Cues to counteract socioeconomic differences? BMC. *Public Health*, 10(1), 154.
33. Glanz, K., Gertraud, M., & Carlin, L. (2005). Ethnicity, sense of coherence, and tobacco use among adolescents. *Annals of Behavioral Medicine*, 29(3), 192–199.
34. Gochman, D. S.(1988). Health behavior **:** emerging research prospectives Plenum Press, *New York*.
35. Hampel, P., Rudolph, H., Stachow, R., & Petermann, F. (2003). Multimodal patient education program with stress management for childhood and adolescent asthma. Patient Education and Counseling, 49(1), 59–66.
36. Haoka, T., Sasahara, S., Tomotsune, Y., Yoshino, S., Maeno, T., & Matsuzaki, I. (2010). The effect of stress-related factors on mental health status among resident doctors in Japan. *Medical Education*, 44(8), 826-834.
37. Hatherill, S. (2007). Psychiatric aspects of chronic physical illness in adolescence：significant numbers of chronically ill adolescents have problems coping with their illness. *Continuing Medical Education*, 25(5), 212-214.
38. [Hoshino, H](http://www.ncbi.nlm.nih.gov/pubmed/?term=Hoshino H%5BAuthor%5D&cauthor=true&cauthor_uid=22688065)., [Izawa, Y](http://www.ncbi.nlm.nih.gov/pubmed/?term=Izawa Y%5BAuthor%5D&cauthor=true&cauthor_uid=22688065)., [Suzuki, N](http://www.ncbi.nlm.nih.gov/pubmed/?term=Suzuki N%5BAuthor%5D&cauthor=true&cauthor_uid=22688065). (2012). Research Committee on Moyamoya Disease. Epidemiological features of Moyamoya disease in Japan. [*Neurologia Medico-Chirurgica (Tokyo)*](http://www.ncbi.nlm.nih.gov/pubmed/22688065), *52*(5): 295-298.

# Hwang Y.S. (2010). Clinical Features of Moyamoya Disease: An Overview. [***Moyamoya Disease Update***](https://link.springer.com/book/10.1007/978-4-431-99703-0)**; 107-109.**

1. Ihm, M. O., Song, M. K., & Kim, C. S. (2012). A mediating model of social support between anger and psychological maladaptation of adolescents. *Korean Journal of Youth Studies*, 19(4), 247-270.

# Ishikawa, T., Tanaka, N., Houkin, K., Kuroda, S., Abe, H., Mitsumori, K. (1998). Regional cerebral blood flow in pediatric Moyamoya disease: Age-dependent decline in specific regions. *Child‘s Nervous System*, 14(8):366-71.

1. Kleinloog, R., Regli, L., Rinkel, G. J., Klijn, C. J. (2012). Regional differences in incidence and patient characteristics of moyamoya disease: a systematic review. *J Neurol Neurosurg Psychiatry*, 83(5), 31-6.
2. Koposov, R. A., Ruchkin, V. V., & Eisemann, M. (2003). Sense of coherence: A mediator between violence exposure and psychopathology in Russian juvenile delinquents. *The Journal of Nervous and Mental Disease*, 191(10), 638–644
3. Kovasc, M. (1985). The Children’s Depression, Inventory (CDI). *Psychopharmacology Bulletin*, 21(4): 995-998.
4. Kovasc, M. (2003). Children‘s depression inventory (CDI) Technical manual update. *Toronto, Canada*: Multi-Health Systems. Pp. 96.
5. Kook, S. H., & Varni, J. W. (2008). Validation of the Korean version of the pediatric quality of life inventory 4.0(PedsQL) generic core scales in school children and adolescents using the Rasch model. *Health and Quality of Life Outcomes,* 2(6), 41.
6. Kim J.S. (2016) Moyamoya disease: epidemiology, clinical features, and diagnosis. *J Stroke*, 18(1):2–11.
7. Kim, H. Y., Chung, C. S., Lee, J., Han, D. H., Lee, K. H. (2003). Hyperventilation-induced limb shaking TIA in Moyamoya disease. *Neurology*. 60(11), 137-9.
8. Kim, J. (2015). Mental Health in Adolescents with Allergic Diseases-Using Data from the 2014 Korean Youth’s Risk Behavior Web-based Study*. J Korean Soc Sch Health*. 28(2), 79-88.
9. Kim, Y.J. (2006). *The relationships among communication in parentchild, stress coping and adolescents’ school adjustment* [master’s thesis]. Seoul: Seoul Women’s University; P. 1-80.
10. Lim, H. S. (2011). *The development and effectiveness of the family resilience enhancement program forthefamily of chronic schizophrenic patient*[dissertation].Seoul:
11. Li, X., Chi, P., Sherr, L., Cluver, L., Stanton, B. (2015). Psychological Resilience among Children Affected by Parental HIV/AIDS: A Conceptual Framework. *Health Psychol Behav Med*, 3(1), 217-235.
12. Malecki, C. K., Demaray, M. K., & Elliott, S. N. (2000). *The Child and Adolescent Social Support Scale*. DeKalb: Northern Illinois University.
13. Masten A. S. (2001). Resilience in children threatened by extreme adversity: Frameworks for research, practice, and translational synergy. Development and Psychopathology. 23(2), 493–506.
14. Mattila, M. L., Rautava, P., Honkinen, P. L., Ojanlatva, A., Jaakkola, S., & Aromaa, Ml. (2011). Sense of coherence and health 21ehavior in adolescence*. Acta Paediatrica,* 100(12), 1590–1595.
15. Moksnes, U. K., Espnes, G. A., & Haugan, G. (2013). Stress, sense of coherence, and emotional symptoms in adolescents. *Psychology & Health*, 29(1), 32–49.
16. Moksnes, U. K., Espnes, G. A., & Lillefjell, M. (2012). Sense of coherence and emotional health in adolescents. *Journal of Adolescence*, 35(2), 433–441.
17. Moksnes, U. K., Rannestad, T., Byrne, D. G., & Espnes, G. A. (2011). The association between stress, sense of coherence and subjective health complaints in adolescents: Sense of coherence as a potential moderator. *Stress and Health*, 27(3), e157–e165.
18. Myrin, B. & Lagerström, M. (2008). Sense of coherence and psychosocial factors among adolescents. *Acta Pædiatrica*, 97, 805–811
19. Neuner, B., Busch, M. A., Singer, S., Moons, P., Wellmann, J., Bauer, U. (2011). Sense of coherence as a predictor of quality of life in adolescents with congenital heart defects: A register-based 1-year follow-up study. *Journal of Developmental & Behavioral Pediatrics,* 32(4), 316–327.
20. Nielsen, A. M., & Hansson, K. (2007). Associations between adolescents’ health, stress and sense of coherence. *Stress and Health*, 23(5), 331–341.
21. Nilsson, K.W., Leppert, J., Simonsson, B., & Starrin, B. (2010). Sense of coherence and psychological well-being: improvement with age. *Journal of Epidemiological and Community Health*, 64, 347 – 352.
22. Park, Y. H., Hyun, H. J., Yu, S. J., & Byen, D. H. (2011). Factors related problem behaviors in high school girls. *Journal of Korean Academy of Community Health Nursing*, 22(3), 315-324.
23. Phi, J.H., Wang, K.C., Cho, B.K., Kim, S.K. (2008). Pediatric cerebrovascular disease. *Korean J Pediatr*. 51(12), 1282-1289.
24. Pelicand, J., Fournier, C., Le Rhun, A., & Aujoulat, I. (2015). Self-care support in paediatric patients with type 1 diabetes: Bridging the gap between patient education and health promotion? A review. Health Expectations, 18(3), 303–311.
25. Pender, N. J. (1996). Health promotion in nursing practice (3^rd^ ed.). Connecticut: *Appleton & Lange Stanford*.
26. Perminas, A, Dovile N. (2012). Changes in the Sense of Coherence of 13-17- Year-Old Adolescents after the Application of Cognitive-Behavioural Intervention. *Socialiniu Mokslu Studijos*. 4(4);
27. Sagy, S., & Braun, L. O. (2009). Adolescents under rocket fire: When are coping resources significant in reducing emotional distress? *Global Health Promotion*, 16(4), 5–15.
28. Sarah, L., Michael, J., Rivkin, A. K., Gabrielle, J. E. (2017). Moyamoya Disease in Children: Results From the International Pediatric Stroke Study. *Journal of Child Neurology*, 32(11), 924-929.
29. Selye, H. (1974). Stress without distress. *Philadelphia, PA*: J.B. Lippincott Co.
30. Scott, R. M., Smith, E. R. (2009) Moyamoya disease and moyamoya syndrome. *N Engl J Med* 360(12), 1226–1237
31. Shim, K. W., Park, E. K., Kim, J. S., & Kim, D. S.(2015), Cognitive Outcome of Pediatric Moyamoya Disease*. J Korean Neurosurg Soc.* 57 (6) , 440-444.
32. Shin, Y. M. & Cho, S. M. (2012). Emotional and behavioral problems in children with chronic physical illness. *Annals of Pediatric Endocrinology & Metabolism*, 17, 1-9.
33. Shin, Y. H., Sim, M. K., & Kim, T. I. (2006). Resilience and Health-Related Quality of Life in Children with Chronic Illness, *Journal of Korean Academy of Child Health Nursing*, 12(3), 295-303.
34. Sivertsen, B., Petrie, K. J., Wilhelmsen-Langeland, A., & Hysing, M. (2014). Mental health in adolescents with Type 1 diabetes: results from a large population-based study. *BMC Endocrine Disorders*, 14:83.
35. Smith, J., Scott, R. M. (2001). Treatment of Moyamoya syndrome in children. *Seminars in Cerebrovascular Diseases and Stroke*, 1(3), 225-239.
36. Su, S. H., Hai, J., Zhang, L., Wu, Y. F., Yu, F. (2013). Quality of life and psychological impact in adult patients with hemorrhagic moyamoya disease who received no surgical revascularization. *Journal of the Neurological Sciences*, 328(1-2), 32-36.
37. Suzuki, J., Takaku, A (1969). Cerebrovascular “moyamoya” disease. Disease showing abnormal net-like vessels in base of brain. *Arch Neurol* 20: 288-299.
38. .
39. Yasargil, M. G., Yonekawa, Y., Denton, I., Piroth, D., Benes, I. (1974). Experimental intracranial transplantation of autogenic omentum majus. *Journal of Neurosurgery,* 39, 213-217.
40. Yeom, I. S., Kim, D. S., Lee, E. Y., Kim, H. S. (2015). The Characteristics of Intellectual and Psychological in the Children with Moyamoya Disease. *Child Health Nurs Res*. 21(2), 123-130.
41. Yeom, I. S., and Oh, W. O. (2018). Development and validation of a scale to measure health behavior of adolescents suffering from moyamoya disease, *undergoing submission*.
42. Yim, S. H., Cho, C. B., Joo, W. I., Chough, C. K., Park, H. K., Lee, K. J (2002). . Prevalence and epidemiological features of Moyamoya disease in korea. *Journal of Cerebrovascular and Endovascular Neurosurgery*, 14(2):75–78.
43. Takanashi, J. I. (2011). Moyamoya disease in children. *Brain and Development*, 33(3), 229-234.
44. The National Youth Policy Institute. Korean youth indicator survey V: Health and safety (protection) [Internet]. Seoul: Author; 2010 [cited 2012 December 31]. Available from: <http://118.128.24.6/pdfs/2010/11.pdf>
45. Varni, J. W., Katz, E. R., Seid, M., Quiggins, D. J. L., and Friedman-Bender, A. (1998a). The Pediatric Cancer Quality of Life Inventory-32 (PCQL-32): I. Reliability and validity. Cancer, 82: 1184–1196.
46. Varni, J. W., Seid, M., & Kurtin, P. S. (2001). PedsQL 4.0: Reliability and validity of the pediatric quality of life inventory version 4.0 genericcore scales in healthy and patient populations. *Medical Care, 39*(8), 800-812.
47. van Dyck, P. C., Kogan, M. D., McPherson, M. G., Weissman, G. R., & Newacheck, P. W. (2004). Prevalence and characteristics of children with special health care needs. Archives of *Pediatrics and Adolescent Medicine*, 158(9), 884-890.
48. Wang, Q., Hay, M., Clarke, D., & Menahem, S. (2014). Associations between knowledge of disease, depression and anxiety, social support, sense of coherence and optimism with health-related quality of life in an ambulatory sample of adolescents with heart disease. Cardiology in the Young, 24(1), 126–133.
49. Wakai, K., Tamakoshi, A., Ikezaki, K., Fukui, M., Kawamura, T., Aoki, R., Kojima, M., Lin, Y., Ohno, Y. (1997). Epidemiological features of moyamoya disease in Japan: findings from a nationwide survey. *Clin Neurol Neurosurg* , 99(S2), S1-5.
50. White, R., Walker, P., Roberts, S., Kalisky, S., & White, P. (2006). Bristol COPD Knowledge Questionnaire (BCKQ): Testing what we teach patients about COPD. *Chronic Respiratory Disease*, 3, 123-131.
51. . Ware, J. E. (1976). Scales for Measuring general health perceptions. *Health Services Research*, 11, 396- 415.
52. Zhao, M., Zhang, D., Wang, S., Zhang, Y., Wang, R., Deng, X., Gao, F., Zhao, J. (2017) Adolescents with moyamoya disease: clinical features, surgical treatment and long-term outcome. *Acta Neurochirurgica*. 159(11), 2071–2080.

**부 록 (도구 사용 허가서)**


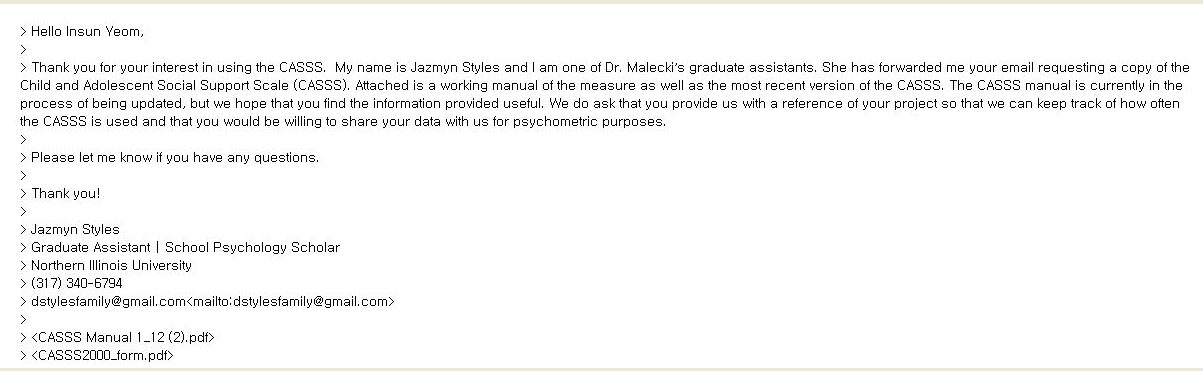


**
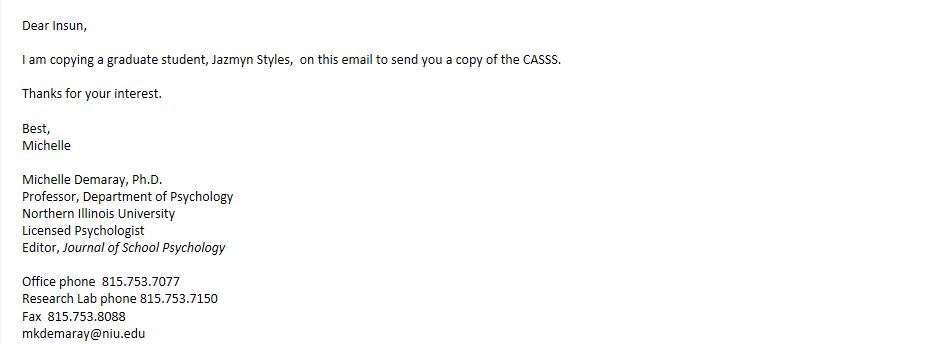
**


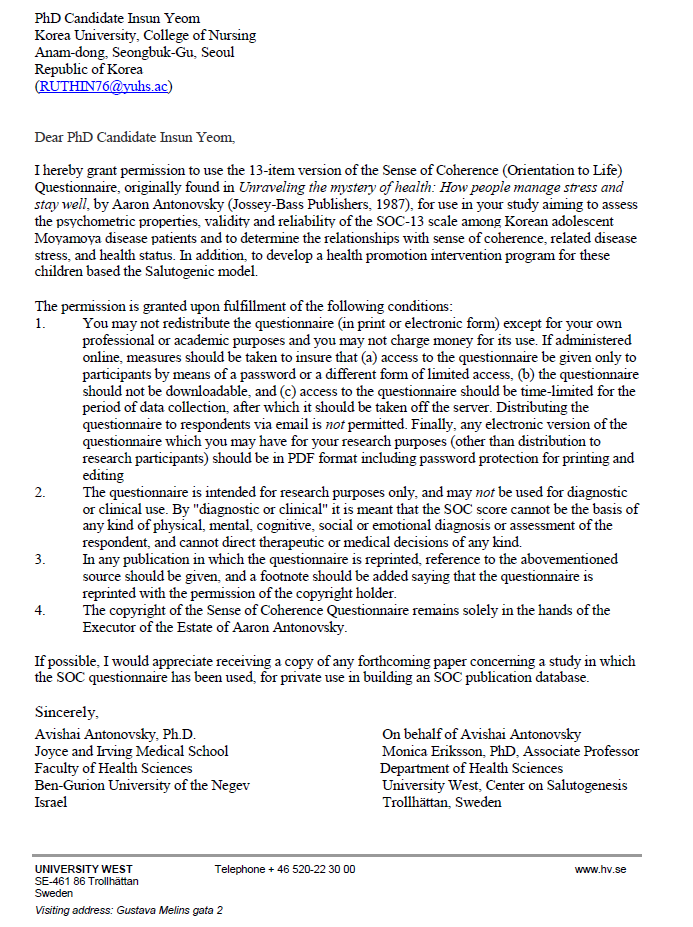

Supplement: S2 File — (DOC) [file pone.0284015.s002.doc]
