# Supplementary material for: Development and effects of salutogenesis program for adolescents with moyamoya disease: A randomized controlled trial
Source: PLoS One. 2023 Oct 26;18(10):e0284015. doi: 10.1371/journal.pone.0284015 (PMC10602295; doi:10.1371/journal.pone.0284015)

**연구계획서**

*****

**1. 연구제목 : 모야모야병 청소년의 건강생성 프로그램 개발 및 효과**

**2. 연구배경**

모야모야병은 뇌내 동맥혈관 말단 부위가 특별한 이유 없이 서서히 좁아지다가 결국 막히면서 뇌의 기저부에 비정상적인 미세 혈관들이 관찰되는 만성 진행성 뇌혈관 폐쇄성 질환으로 희귀 난치성 질환으로 분류되어있다(Suzuki, 1969; Kim, 2016). “Moyamoya” 라는 이름은 일본어로 “담배 연기 모양” 처럼 뇌혈관 영상에서 막힌 부분을 보완하기 위해 형성된 작은 혈관의 희귀한 모양을 묘사하여 붙여진 이름이다. 모야모야병은 1957년 첫 증례가 보고 된 이후 1960 년대 일본에서 처음으로 기술되었으며, 그 후 전 세계 다른 국가의 사람들에게서도 보고되었다. 유럽이나 북미보다 아시아 국가에서 발생률이 높고, 유병률은 점점 증가 추세이다. 한국의 모야모야병 유병률은 2004년 인구 10만명당 6.3명에서 2008년 9.1명, 2011년에는 인구 10만명당 16.1명, 2015년에는 18.1명으로 증가하였고(Kim, 2016), 새로 발생하는 환자의 발생 추이를 보면 2004년에서 2008년까지 매년 약 15%씩 증가하였다(Yim et al., 2012). 또한 2007년부터 2011년까지 매년 발병률은 인구 10만명당 1.7명에서 2.3명으로, 가장 최근 조사에서는 4.3명으로 증가하였다(Kim et al, 2016). 남녀 비율은 2008년 1:1.98에서 2015 년에는 1:2.1 명으로 여성에게서 더 많은 발병률을 보이고 있다. 연령별 유병률은 10-19세 21.6%, 50-59세 30% 로 주로 청소년기와 성인기 후기에 호발하는 것으로 알려져 있다. 전체 모야모야병 중에 가족력이 있는 경우는 10~15%이고 환자의 부모나 형제의 발병 위험도는 일반 인구에 비해 30배에서 40배 높다(Kim et al, 2016).

모야모야병의 원인에 대하여 여러 연구가 꾸준히 진행되어 모야모야병 관련 유전자 연구에서 감수성 유전자를 확인하였지만(Fujimura et al, 2014) 그 발병기전이나 병인을 완전히 정의하지는 못하고 있다. 현재, 모야모야병의 임상적 중재 수술법으로 완치가 아닌 증상의 경감이나 뇌경색, 뇌출혈의 위험을 감소시키는 정도의 기대 효과를 가지고 있는 정도이기 때문에(Takanashi, 2011), 모야모야병을 가진 환자들의 일상생활에서의 건강행위가 매우 강조된다.

모야모야병의 주요 임상 증상은 아동, 청소년과 성인이 다소 차이가 있다 즉, 아동과 청소년은 반복적인 일과성 뇌허혈 발작(transient ischemic attack, TIA)이 흔하고 성인에서는 뇌출혈이 흔하다(Wakai et al, 1997; Kleinloog et al, 2012). 뇌허혈성 발작은 뇌혈류의 공급 장애로 나타나는 구체적 증상으로 반신마비, 감각이상, 언어장애, 경련발작, 고차원적 뇌기능 장애 및 시력장애 등이 일시적 혹은 영구적으로 있다(Bang, Fujimura, & Kimc, 2016; Bersano et al.,2016).

임상적 증상의 촉발 요인에는 노래 부르기, 악기 불기, 심한 울음, 과도한 운동, 뜨겁거나 매운 음식 먹을 때와 같은 과호흡을 동반한 상황이 관련된다(Kim et al, 2016). 모야모야병에서 과호흡이 일과성 허혈발작을 일으키는 기전은 과호흡이 혈중 이산화탄소 농도를 감소시키고, 이로 인하여 뇌혈관 수축이 일어나 혈액순환이 부족한 모야모야 병변 부위에 일시적인 뇌허혈이 발생하게 된다. 이와 관련하여 모야모야병 아동 또는 청소년들의 과거 증상을 들어보면, 부모에게 혼난 후 심하게 울다가 허혈 증상이 나타나는 경우가 있는데, 대개 아동의 심리적인 반응으로 생각하고 전문가를 찾지 않았음을 알게된다. 일과성 뇌허혈 발작은 대개 아동이 스트레스를 받는 상황에서 발생되는데 몇 초에서 몇 분 이내에 증상이 바로 좋아지지만 지속 시간이 길어질 경우 뇌허혈에서 끝나지 않고 뇌경색으로 진행할 수 있기 때문에 일상적 생활에서의 스트레스 관리를 위한 건강행위가 요구된다(Hwang, 2010). 이러한 질병 관련 뇌허혈, 뇌경색 및 뇌출혈을 겪게 되면, 심리적, 신체적, 정신적 부담감이 높아져, 이로 인한 개인의 삶의 질 저하가 두드러지게 된다(Su et al., 2013).

모야모야병을 가진 청소년은 임상적 증상과 함께 그들의 발달과정상 겪게 되는 다양한 심리적 도전과 맞물려 정상 청소년에 비해 개인의 삶의 질이 감소된다(Shim et al, 2015). 이는 청소년 시기가 아동기에서 청년기로 넘어가는 과도기로 신체적, 정신적 측면에서 급격한 변화를 겪고 사춘기의 이차적 성징의 발달과 아울러 신체 발달에 따른 심리적 경험과 미래 삶의 대한 틀이 형성되는 중요한 시기이기 때문이다. 따라서 이런 정상적인 발달 단계에서 경험하는 심리적, 사회적 변화에 잘 적응하지 못하는 경우에는 신체상의 문제나 사회적인 문제와 연관하여 문제를 일으킬 수 있는데, 모야모야병과 같은 만성진행성 질환의 발병은 청소년의 정상적인 성장발달에 매우 부정적인 영향을 미친다(김윤정 & 권혜진, 2013).

또한 청소년기에 경험하는 신체적 질환은 그들에게 상당한 스트레스로 작용할 수 있는데(Chmlin & Chren, 2010), 모야모야병으로 지속적인 내과적 치료 및 수술 등을 받게 되는 청소년들은 신체적 문제와 함께 심리, 사회적 어려움을 경험할 가능성이 높다.

만성질환을 가진 청소년이 경험하는 신체적 제한, 정기적인 치료 및 검진과 관련하여 많은 경우 통증과 두려움을 경험하고 있으며, 특히 급격한 신체적, 심리적, 사회적 변화를 경험하는 청소년에게 있어 질병 그 자체가 스트레스 요인으로 작용할 수 있다(Shin, Sim, & Kim, 2006). 스트레스는 긴장(tension)을 유발하며, 긴장을 성공적으로 해결하지 못할 경우 신체적, 정신적 압박이 지속되면 질병의 악화를 초래할 수 있다(Lazarus & Folkman, 1984). 특히 모야모야병 청소년은 병의 진행 과정 상에서 과호흡 또는 스트레스 상황에서 경험하는 뇌허혈 증상으로 심리적 위축과 신체적 활동에 제한을 경험하게 되며 또래 친구들처럼 학교생활을 정상적으로 해내지 못하는 것에서 오는 심리적 위축은 개인의 스트레스를 성공적으로 해결하지 못하게 만든다. 이러한 스트레스가 반복될 경우, 모야모야병의 주증상인 허혈성 증상의 경험이 더 빈번하게 발생되는 악순환이 초래되기 때문에, 모야모야 청소년이 수행해야할 건강행위 중 스트레스 관리는 매우 중요하다. 이는 스트레스 요인에 의한 긴장을 효과적으로 해결하지 못할 경우 개인의 건강행위 뿐만 아니라 우울이나 불안과 같은 내재화 문제 행동에도 영향을 미치게 되기 때문이다(Ihm, Song, & Kim, 2012; Park, Hyun, Yu, & Byen, 2011). 이처럼 모야모야병과 같은 만성질환 가진 청소년의 부정적 정서는 직접적으로 주관적인 삶의 질에 영향을 주고 있기 때문에([Anna](https://www.ncbi.nlm.nih.gov/pubmed/?term=Zashikhina A%5BAuthor%5D&cauthor=true&cauthor_uid=24460738) and [Bruno,](https://www.ncbi.nlm.nih.gov/pubmed/?term=Hagglof B%5BAuthor%5D&cauthor=true&cauthor_uid=24460738) 2014), 근본적으로 그들의 스트레스 관리는 우울이나 불안과 같은 부정적 정서의 초래를 최소화 할 뿐만 아니라, 더 나아가 그들의 삶의 질 증진을 위해서도 매우 중요하다.

모야모야병 청소년들이 겪고 있는 어려움이나 스트레스를 관리하고 긍정적인 방향으로의 건강한 삶을 이끌기 위해 적용해 볼 수 있는 이론으로 Antonovsky(1979)가 제시한 건강생성이론을 제시할 수 있다. 건강생성이론(Salutogenesis theory)은 개인에게 내∙외적 스트레스요인이 될 수 있는 질병요인이나 긴장유발요인에 대한 성공적인 적응을 통해 건강에 이르는 과정에 초점을 두고 있다. 이 이론에서는 건강과 질병을 분리하는 전통적인 의학모델인 이분법을 부정하고, 건강과 질병은 안락함과 불편함의 연속선상에 위치하는 것으로 인간은 균형회복과정에서 항상 이리저리 움직이고 있다고 하였다(Antonovsky, 1979). 즉 건강생성이론의 관점에서 보면, 건강이란 동일한 축에 놓여 있는 안락함과 불편함 사이를 오가는 것이고, 무엇이 사람을 안락함의 방향으로 움직이게 해주는거를 논의하고 있다.

Antonovsky(1979)에 의하면 인간은 환경과 활발하게 상호작용하는 개방체계(open system)로서 외부환경과의 상호작용 과정에서 내∙외적 자극에 의하여 긴장과 압박감이 발생할 수 있다. 그러나 이 긴장과 압박감은 잠재적 질병 발생인자가 아닌 건강을 촉진하는 인자일 수도 있다고 설명하였다. 이러한 환경은 개인에게는 스트레스 요인임과 동시에 저항자원이 될 수 있는데, 건강생성이론에서는 개인이 가지고 있는 잠재적인 혹은 실제적인 일반적 저항자원(Generalized Resistance Resource)과 이 자원을 적절히 파악하고 사용, 관리할 수 있는 통합력(Sense of Coherence)을 핵심 개념으로 설명하고 있다. 즉 일반적저항자원과 통합력은 구체적으로 개인의 건강을 위협하는 위험인자들을 최소화할 뿐만 아니라 개인의 문제를 적절히 해결하게 하는 것으로서 이 이론의 핵심 개념이다(Antonovsky, 1987).

이 이론의 첫번째 요소인 일반적 저항자원은 스트레스 요인에 의한 긴장을 해결하고 통합력을 동원하도록 하는 자원을 의미한다. 두번째 요소인 통합력은 일반적 저항자원과 연결되어 스트레스에 성공적으로 적응할 수 있도록 직접적으로 문제를 해결 하는 능력을 의미한다(Antonovsky, 1987). 즉, 건강생성이론은 개인이 삶의 경험에서 피할 수 없는 스트레스와 질병요인에도 불구하고 개인의 내적 능력과 자원에 의해 스트레스를 능동적으로 극복하여 건강에 이르는 과정을 설명한다. 따라서 모야모야병을 가진 청소년 역시 그들의 신체적, 인지적 제한에도 불구하고 그들이 활용 가능한 일반적 저항자원을 효율적으로 동원하여 통합력을 발휘하도록 하는 건강생성이론의 적용은 모야모야병 청소년의 건강생성과 살의 질 향상을 위해 유용할 것으로 판단된다.

현재까지 모야모야병 청소년을 대상으로 진행한 선행연구는 매우 제한적이다. 국외의 경우 모야모야병 청소년의 수술적 중재를 시행한 후 임상적 증상의 변화에 관한 장기 결과 분석 등(Zhao et al, 2017; Bao, 2015)이 확인되었고, 국내의 경우 모야모야병 청소년의 임상적 증상에 관한 연구와 소아 청소년의 인지기능, 정서적 특성에 관한 조사 연구 등(이승익 외, 2007; Yeom et al, 2015)만이 확인되고 있다**.** 또한 본 연구에서 고려하고 있는 건강생성이론을 기반으로 모야모야병에 적용하여 연구를 수행한 것은 발견하지 못하였고, 다만 건강생성 이론의 하위요인인 통학력 증진에 초점을 둔 연구들이 소수 진행된 바 있다. 가령, 선천성 심장병, 당뇨병 등의 만성 질환 청소년을 대상으로 통합력을 증진시키기 위한 교육 프로그램의 중재 후 대상자들의 스트레스에 대한 대처를 높이고, 일상생활 적응행동과 삶의 질 증진에 긍정적인 효과가 있음을 보고하였다(Apers, et al., 2013; Pelicand, et al, 2015; Hampel, et al, 2003).

위의 내용을 정리하면 다음과 같다. 모야모야병은 질병의 특성상 만성적 진행적 특성이고, 특히 모야모야병을 지닌 청소년들은 정상적 발달 과정에서 겪는 다양한 신체적, 심리적 변화에 부가적으로, 질병 치료과정으로 인한 일상생활 유지의 어려움에 따른 고립과 부정적인 정서적 경험 및 예측 불가능한 뇌허혈 증상으로 스트레스를 받고 있으며, 나아가 삶의 질 저하를 경험하고 있다. 이에 모야모야병 청소년에게 건강생성이론에 근거한 중재의 적용은 스트레스를 유발하는 근본적인 모야모야병을 제거하려는 시도보다는 자신이 스트레스를 극복할 수 있도록 하는 개인적 역량을 증진시켜 줄 수 있을 것이다.

따라서 본 연구에서는 기존의 연구들이 건강생성이론의 일부만을 적용하여 중재를 시도한 것과 달리 건강생성이론이 두가지 하위 개념 모두를 적용한 건강생성 중재 프로그램을 개발하고 그 효과를 확인하고자 한다.

**3. 연구목적**

본 연구에서는 모야모야병 청소년 대상 건강생성 프로그램 개발하고 그 효과를 검정하기 위한 것으로 구체적인 연구 목적은 다음과 같다.

1) 모야모야병 청소년의 건강향상을 위한 건강생성(Salutogenic) 프로그램을 개발한다.

2) 모야모야병 청소년의 건강향상을 위한 건강생성(Salutogenic) 프로그램의 효과를 검증한다.

**4. 연구 실시 기관명 및 주소**

세브란스 어린이 병원, 서울시 서대문구 연세로 50-1

**5. 연구 지원기관**

없음

**6. 연구책임자, 공동연구자, 담당자의 성명과 직명**

| **세부** | **성 명** | **소 속** | **직위** | **구 분** | **전공 및 학위** | | | |
| --- | --- | --- | --- | --- | --- | --- | --- | --- |
|  |  |  |  |  | **학위** | **연도** | **전공** | **학교** |
|  | 심규원 | 신경외과 | 부교수 | 내부 | 박사 | 2010 | 신경외과학 | 연세대학교 |
|  | 김동석 | 신경외과 | 교수 | 내부 | 박사 | 2002 | 신경외과학 | 인하대학교 |
|  | 염인선 | 신경외과 | 연구담당 | 내부 | 석사 | 2006 | 간호과학 | 연세대학교 |

**7. 연구기간**

- 연구 소요 예상 기간(IRB 승인일로부터 ~ 12개월)

**8. 연구대상자**

본 연구의 연구대상자 선정은 서울소재 세브란스 병원 모야모야병 클리닉 외래방문 혹은 입원한 모야모야병 진단 받은 자로 본인 및 법정대리인이 본 연구의 목적을 이해하고 서명 및 동의한 자, 설문지를 읽고 응답할 수 있으며 의사소통이 가능한 자를 대상으로, 1,2 단계의 구체적인 선정기준 및 제외기준은 다음과 같다.

**1단계**

- 본 연구 대상자의 구체적인 선정기준은 다음과 같다.
- 1) 만 13세에서 만18세 청소년, 청소년의 부모
- 2) 모야모야병 진단 받은 지 1개월 이상 된 자

본 연구 대상자의 구체적인 제외기준은 다음과 같다.

- 의무기록상 정신질환 병력이 있거나, 프로그램개발의 질에 지장을 주는 질환(예, 청각장애, 시각장애 등)이 있는 자

**2단계**

- 본 연구 대상자의 구체적인 선정기준은 다음과 같다.

1. 모야모야병 진단 받은 지 1개월 이상 된 자
2. 프로그램 검증을 위해 2단계 연구에 참여 가능한 자

- 본 연구 대상자의 구체적인 제외기준은 다음과 같다.
- 의무기록상 정신질환 병력이 있거나, 프로그램에 지장을 주는 질환(예, 청각장애, 시각장애 등)이 있는 자
- 대상자의 실험군과 대조군의 배정은 그룹간의 혼돈변수를 통제하기 위해서 연구에 참여한 순서에 따라 Random number generator ([http://randomizer.org](https://email.yuhs.ac/owa/redir.aspx?C=kdhBRnJoAH_SMV6_BSLC7UEx0buDZ_KZ2AxkV9NR_H2qLZVyAK_VCA..&URL=http%3A%2F%2Frandomizer.org))을 이용하여 각각 무작위배정(randomization) 할 예정이다 .

   무작위 할당은 컴퓨터 프로그램을 이용하여 전체 대상자 수만큼 난수를 생성한 후 중앙값을 기준으로 1과 2로 구분하고, 이후 연구에 참여한 순서에 따라 대상자를 각 집단으로 무작위 배정할 예정이다.

등 록

선정조건 평가 (N= )

제외 (N= )

-거부( N= )

-제외기준(N= )

무작위 배정(N= 60 )

할 당

실험군 배정 (N=30 )

대조군 배정 (N=30 )

사후 조사

연구 도중 탈락 (N= )

연구 도중 탈락 (N= )

분 석

분석 (N= )

분석 (N= )

<그림 1> CONSORT diagram

- **취약한 대상자 모집 시, 보호 방안**

1. 모야모야 질환 청소년 환아는 뇌혈관 질환의 미성년으로 의사소통이 가능한 대상의 경우, 자연스러운 분위기에서 이 연구에 대하여 가능한 쉬운 언어로 설명하고 동의를 구한다.

2. 부모님 중에 가능한 한 분에게 충분한 설명을 하고 연구에 대한 동의를 취득한다. 부모님이 계시지 않는 경우는 법적인 보호자에게 동의를 취득한다.

3. 연구 참여자의 의지에 따라 언제라도 동의를 철회할 수 있고, 이에 따른 치료에 대한 어떠한 불이익도 받지 않는다.

**9. 예상 연구대상자 수와 산출 근거**

1단계에서는 심층면담 모야모야병 청소년 6명, 부모 6명.

2단계에서의 연구대상자 수 산출은 G*power 3.1.9.2를 이용하였으며, 연구의 효과크기는 선행 연구 중 Perminas & Dovile(2011)이 청소년을 대상으로 통합력 증진을 위해 적용한 중재 프로그램 연구 결과에서 보고된 통합력 점수를 이용하였다. 이 연구에서  실험군(M=45.6, SD=2.8)과 대조군(M=43.3, SD=3.4)의 결과를 이용하여 산출된 효과크기(d)는 0.74 였으며, 유의수준 α= .05, 검정력(1-β)=.80으로 산출한 결과 집단별 대상자 수는 각각 24명이었다. 탈락률 25% 고려하여 총 실험군, 대조군을 각각 30명, 총 60명을 모집할 예정이다.

연구 담당자가 직접 동의를 구하여 모집할 예정이다.

**10. 연구대상자 모집**

세브란스 병원 소아신경외과 또는 신경외과에서 모야모야 질병 진단 받고 외래 혹은 입원한 환자 만 13세에서 18세에 해당하는 대상자 및 보호자에 대하여, 연구의 필요성에 대해 설명하고 이에 대한 서면 동의를 진행할 예정이다. 청소년 대상은 취약 대상군으로 대상자 및 보호자 동의가 함께 이루어진 경우에만 연구에 참여하도록 할 예정이다.

**11. 연구대상자 동의 및 연구 참여에 대한 지속적 참여**

연구대상자의 서면 동의를 얻기 위하여 연구 담당자가 직접 연구의 목적, 방법에 대한 설명후 동의서를 직접 받을 예정이다. #8번과 같이 취약한 대상자 모집시 보호 방안의 방법에 따라 진행할 예정이다. 또한 연구 기간동안 지속적 참여에 대한 연구 담당자가 연구 참여 전에 재 확인 예정이며, 대상자 혹은 대상자의 보호자가 철회를 원하실 경우 연구에서 제외하도록 할 예정이다.

**12. 구체적 연구방법**

본 연구는 모야모야병 청소년의 건강증진을 위한 모야모야병 건강생성 프로그램을 개발하는 연구(1단계)와 개발된 모야모야병 건강생성 프로그램의 효과를 검정하기 위한 실험연구(2단계)로 구성할 예정이다<그림 2>.

구체적으로 프로그램을 개발하기 위하여 기획단계, 프로그램 구성단계, 예비연구 단계, 프로그램 실시 및 평가 단계를 적용한 김창대 등(2011)의 프로그램 개발 모형을 참고할 예정이다.

기획단계는 문헌고찰과 잠재적인 프로그램 수혜자, 즉 모야모야병 청소년을 위한 요구조사를 통하여 프로그램의 목표를 정립하는 단계이다. 본 연구에서는 프로그램의 대상자인 모야모야병 청소년이 자신이 가지고 있는 문제에 집중하기 보다는 자신에게 잠재되어있는 자원에 집중하여 현재 자신이 가지고 있는 저항자원을 인식 및 향상 시키는 것을 목표로 한다. 또한 자신의 삶을 체계화하고 자원을 관리 할 수 있는 통합력을 증진시키기 위한 것으로 관련 문헌 고찰을 진행할 예정이다. 더불어 청소년, 부모 및 임상 전문가 중심으로 심층 면담 및 포커스그룹 인터뷰를 실시할 예정이다.

구성단계는 기획단계에서 얻어진 결과를 중심으로 목표달성을 위해 프로그램의 구성요인, 구성내용, 운영방법 등을 도출하고 각각의 원리에 부합되는 프로그램의 주요내용을 구성하여 각 요소를 조직하는 단계이다. 본 연구에는 기획단계의 문헌고찰 및 임상 전문가와의 포커스그룹 인터뷰 결과를 토대로 모야모야병 청소년기의 특징을 고려함과 동시에 일반적 저항자원과 개인의 통합력을 증진시키는 프로그램의 구성과 내용을 조직할 예정이다.

마지막으로 프로그램 실시 및 평가단계에서는 구성단계에서 얻어진 프로그램 초안을 소수의 실험적 대상에게 실시하여 프로그램의 효과를 측정하고, 참여자들로부터 평가적 의미의 피드백을 받게 된다. 이를 통해 프로그램의 활동, 내용, 전략 등을 수정, 보완하여 구성된 프로그램의 일반화를 위해 프로그램의 완성도를 높이는 단계라고 할 수 있다. 이후 전문가의 감수와 자문을 통해 수정, 보완을 거친 후 최종적인 프로그램을 개발할 예정이다. 본 연구에 적용할 프로그램 개발절차 단계별 내용은 <그림 2> 와 같고 세부적 내용 (안)은 다음과 같다.

기

획

목표 수립

프로그램 목표 수립

**1단계**

문헌 연구

모야모야병 청소년/만성질환 청소년의 특성 관련 선행연구

건강생성 이론 적용 선행연구 고찰

모야모야병 청소년 개별 심층 면담,

전문가, 모야모야병 청소년 부모 포커스 그룹 인터뷰

요구 분석

구

성

모형 개발

프로그램 구성요인 및 내용 추출

프로그램의 운영방법 추출

프로그램 요소 조직 및 활동 내용 구성

임상 전문가의 안면 타당도 평가

프로그램 초안 개발

내용 구성

실시 및 평가

예비 연구

프로그램 시범운영 실시

**2단계**

평가 및 수정, 보완

프로그램 효과성 분석

전문가 자문에 따른 수정 보완

개발 완료

최종 프로그램 개발

<그림 2>. 모야모야병 청소년을 위한 건강생성 프로그램 개발절차 모형(안)

**1단계 : 모야모야병 건강생성 프로그램 개발**

모야모야병 청소년의 건강증진을 위한 모야모야병 건강생성 프로그램 개발은 모야모야병 진단 후 치료 과정과 병의 진행과정에서 요구되는 중재내용을 파악하기 위해 관련 문헌고찰과 모야모야병 청소년 개별 심층 면담과 전문가 그룹과 모야모야병 청소년 부모 그룹에 대한 포커스 그룹 인터뷰 과정으로 진행할 예정이다.

1. **문헌고찰**

모야모야병 청소년의 건강생성을 위해 요구되는 중재 내용을 확인하기 위해 먼저 모야모야병 청소년 대상 중재내용과 본 연구의 이론적 기틀인 건강생성이론의 핵심 개념, 즉 일반적 저항자원과 통합력을 중심으로 이루어진 청소년 대상 중재 프로그램 개발 관련 국내∙외문헌을 고찰하였다, 그 결과는 아래와 같다.

문헌 검색은 PubMed, Google scholar, CINAHL, KoreaMed, RISS 이며 최근 20년간 간행된 논문과 실무 가이드라인을 검색하였다. 검색의 주요용어는 ‘ 모야모야병’, ‘중재프로그램‘, ‘건강생성이론 프로그램’, “salutogenic program‘, ‘Salutogenesis application in adolescent’로 하여 모야모야병 청소년의 건강증진 관련 중재내용을 고찰하였다.

그 결과, 현재까지 모야모야병 관련하여서는 질환의 개념과 치료, 생활 관리, 뇌기능 내용을 확인하였으며, 건강생성이론 바탕의 청소년 중재내용에서는 일반적 저항자원의 인지적 요인으로서 지식적 중재, 정서적 요인으로서 자기 인식, 정서적 친밀감, 스트레스 상황에서의 긍정적 방어방법과 합리적이고 유연하고 통찰력 있는 극복 전략 계획, 사회적 자원으로서 사회적 지지 자원소개와 사회적 네트워크 소개 등에 대한 제안 등이 파악되었다. 또한 통합력 영역에는 구체적 접근으로 이해력, 관리력, 의무부여 등이 포함되어 있는 것으로 파악되었다. 첫째, 이해력은 자신의 내·외적 환경은 자신의 일생을 통하여 예측가능하고, 설명가능하며, 또한 일관되어 있다는 이해를 포함한 인지적 요소이다. 문헌고찰을 통해서 본 이해력의 중재 내용으로는 개인의 현실 사건들을 예측가능하고 합리적으로 벌어진다는 가정 하에 연구자의 탐색적 질문을 통해 개인의 스트레스 상황을 예측하여 보고 설명을 유도함으로써 이해력을 증진시키는 것을 파악하였다. 둘째, 관리력은 환경의 여러 자극에 의해 제기되는 요구들을 만족시키기 위해 가능한 자원을 활용하는 능력으로 구체적 환경적 자극에 의한 문지들을 해결하는데 필요한 자원들을 항시 확보가 가능하다는 믿음을 갖게 하는 것으로 구체적 자원 사용으로는 개인의 기능뿐 아니라 가족, 친구, 종교지도자, 의료인 등의 자원을 통하여서도 사용에 대한 소개를 확인하였다. 셋째, 의미부여는 내·외적 환경으로부터의 요구들이 도전하고, 집중하며, 투자할 가치가 있다고 긍정적으로 받아들이는 능력으로 개인의 인생은 그 자체가 의미있는 것이므로, 질병이나 스트레스와 같은 긴장상태를 회피하기 보다는 문제 해결을 위해 노력을 들여 도전하고, 그 노력이 개인의 인생에 가치가 있는 것을 상담한 내용 등이 확인되었다.

1. **개별 심층면담**

본 연구에서 개별 심층면담을 위한 연구 참여자는 모야모야병 청소년을 대상으로 진행 할 예정이며, 더 이상 새로운 개념이나 내용이 도출되지 않을 때까지 수행할 것이며, 모야모야병 진단을 받은지 1개월 이상된 청소년 6명으로 예상하고 있다(신혜란, 2007).

개별 심층 면담은 20xx년 x월 x일부터 20xx년 x월 x일까지 x일간 서울 특별시 세브란스 병원 모야모야병 클리닉에 방문하는 모야모야병 진단받은 청소년으로 본 연구의 목적과 방법에 대한 설명을 듣고 참여에 동의한 자를 대상으로 진행할 예정이다.

면담진행은 대상자의 시간을 고려하여 미리 면담일시를 정한 후 모야모야병 클리닉 상담실 또는 입원 기간에는 병실에서 주위 환경이 조용하고 편안하게 대화가 용이한 곳으로 할 예정이다. 면담의 시작은 도입부에서 인구학적 특성을 묻는 것으로 시작하여 면담 시 이용할 질문은 다음과 같다.

‘모야모야병 청소년들이 가지고 있는 스트레스를 발생시키는 것들은 무엇이라고 생각하십니까?’

‘모야모야병 청소년들이 스트레스를 성공적으로 대처하기 위해 필요한 개인적인 요인들이 있다면 무엇이 있을까요?’

‘모야모야병 청소년들이 개인 상태의 포괄적 이해를 통해 자신에게 직면한 상황 또는 사건의 해결하는 것에 도움을 줄 수 있는 중재 프로그램이 무엇이 있을까요?’

‘모야모야병 청소년들이 살아가면서 필요한 내적, 외적 자원을 적절히 활용할 수 있다고 느끼는 것이 개인의 삶의 질에 영향을 준다는 면에서 필요한 중재 프로그램이 무엇이 있을까요?’

‘모야모야병 청소년들이 자신 스스로 삶의 의미를 찾아가는 동기적 요소가 성공적 스트레스 대처에 영향할 수 있다고 볼 때, 도움을 줄 수 있는 중재 프로그램 내용에는 어떤 것이 있을까요?’

‘모야모야병 청소년들의 물리적, 사회적 자원, 대처 전략, 자아 존중감 등을 증진시킬 수 있는 중재 프로그램 내용으로는 무엇이 있을까요?’

‘중재 프로그램을 진행한다면 얼마나 자주, 몇 회 정도 해야 한다고 생각하십니까?’

‘모야모야병 청소년에게 있어서 건강한 삶이란 무엇이라고 생각하십니까?’

‘중재 프로그램에서 가장 중점을 주어야 하는 주제는 무엇이라고 생각하십니까?’

‘이야기하고자 한 것 중 하지 못한 것이나, 더 보충할 것이 있으면 말씀해 주십시오.’

평균 면담시간은 40분에서 60분으로 할 예정으로, 면담의 내용은 연구대상자의 동의하에 녹음을 할 예정으로 면담 중 중요한 내용을 메모하겠다. 면담 종료 시에는 연구 참여에 대한 감사의 표시로 모야모야 청소년에게 필요한 5000원상당의 물통을 제공할 예정이다.

1. **포커스 그룹 인터뷰**

본 연구에서 포커스 그룹 인터뷰를 위한 참여자는 전문가 그룹과 모야모야병 청소년 부모 그룹으로 진행할 예정이다. 전문가 그룹 입터뷰를 위한 참여자는 연구자가 원하는 전문성을 가진 개인을 추출하는 의도적 표본 추출법을 이용할 예정이다. 모야모야병 임상 전문가의 기준은 모야모야병 클리닉에서 진료하고 있는 신경외과 전문의 3명과 클리닉에서 전문간호사 경력이 3년 이상인 전문간호사 2명, 클리닉에서 심리검사와 치료를 시행한지 3년이상된 심리학자 1인, 소아신경외과 병동에서 모야모야병 간호를 경험한지 3년 이상된 간호사 6인으로 2개의 그룹으로 2회 수행 할 예정이다. 또한 부모 포커스 그룹 인터뷰는 모야모야병 진단을 받은지 1개월 이상된 청소년의 부모로 3명 혹은4명으로 구성된 2그룹으로 예상하고 있다. 연구자가 연구 참여자들에게 연구 목적을 설명한 후 연구 참여 의사를 최종 확인 후 진행할 예정이다. 연구 참여자들은 자료 수집 전 연구의 목저, 절차, 포커스 그룹 인터뷰의 주요 질문 내용, 자료수집방법(기록, 녹취), 자료 분석 후 이용계획, 자료보관 계획, 연구 참여의 위험성, 보상 등이 기재된 동의서를 읽고 서명 후 진행할 예정이다.

포커스 그룹 인터뷰 진행은 참여자의 시간을 고려하여 미리 인터뷰 일시를 정한 후 어린이병원 회의실에서 주위 환경이 조용하고 편안하게 대화가 용이한 곳으로 할 예정이다. 인터뷰 시 이용할 질문은,

‘모야모야병 청소년이 가지고 있는 스트레스원은 무엇이라고 생각하십니까?’

‘모야모야병 청소년이 스트레스를 성공적으로 대처하기 위해 필요한 개인적인 요인들이 있다면 무엇이 있을까요?’

‘모야모야병 청소년들이 자신에게 직면한 상황 또는 사건의 해결하는 것에 도움을 줄 수 있는 어떤 종류의 프로그램이 필요할까요?’

‘모야모야병 청소년들이 살아가면서 필요한 내적, 외적 자원을 적절히 활용할 수 있다고 느끼는 것이 개인의 삶의 질에 영향을 준다는 면에서 필요한 중재 프로그램이 무엇이 있을까요?’

‘모야모야병 청소년이 자신 스스로 삶의 의미를 찾아가는 동기적 요소가 성공적 스트레스 대처에 영향할 수 있다고 볼 때, 도움을 주기 위해 필요한 어떤 종류의 지지나 훈련이 필요할까요?’

‘모야모야병 청소년의 물리적, 사회적 자원, 대처 전략, 자아 존중감 등을 증진시킬 수 있는 중재 프로그램 내용으로는 무엇이 있을까요?’

‘모야모야병 청소년 삶에서 건강한 삶이란 무엇이라고 생각하십니까?’

‘중재 프로그램을 진행한다면 얼마나 자주, 몇 회 정도는 해야 한다고 생각하십니까?’

‘중재 프로그램에서 가장 중점을 주어야 하는 주제는 무엇이라고 생각하십니까?’

‘이야기하고자 한 것 중 하지 못한 것이나, 더 보충할 것이 있으면 말씀해 주십시오.’

- **모야모야병 청소년의 건강생성 프로그램의 구성 및 내용(안)**

현재까지 문헌고찰을 통해 구성된 모야모야병 청소년의 건강생성 프로그램의 구성요인 및 운영 방법(안)<표 1>, 교육 회기별 프로그램의 구성요소 및 내용(안)<표 2>는 아래와 같다. 향후 모야모야병 청소년과 부모 대상 심층면담, 임상 전문가 포커스 인터뷰 결과를 분석하여 최종 완성할 예정이다.

1. **프로그램 중재내용 구성 요인 및 운영방법(안)**

본 연구의 프로그램은 건강생성이론의 핵심개념인 일반적 저항자원과 통합력 개념을 중심으로 각 개념에 따른 구성요인과 운영전략을 계획하였다.

건강생성 이론에 근거한 모야모야병 청소년 건강생성 프로그램은 자신의 질병과 관련된 증상이나 문제들을 제거하기보다는 자신의 일반적 저항자원에 집중하여 개인이 가지고 있는 저항자원을 재발견하고 증진시키는 것이다. 또한 자신의 일상생활을 체계화하는 것에 자신감을 갖고, 개인의 자원을 관리할 수 있으며, 자신의 삶에 가치 있는 의미를 부여할 수 있는 통합력을 증진시키기 위한 것이다.

1. **일반적 저항자원**

일반적 저항자원은 스트레스원에 의해 나타나는 긴장을 효과적으로 대처하고 개인의 통합력을 동원하도록 하는 자원을 말하는 것으로 개인이 가지고 있는 신체적, 생화학적, 물질적, 인지적, 정서적, 윤리적, 인간관계적, 사회문화적 요소를 포괄하는 개념이다(Antonovsky, 1987). 본 연구에서는 일반적 저항자원을 인지적, 정서적, 사회적 자원 요인으로 사회적 요인은 인간관계적, 사회문화적 요소를 말한다.

1. **인지적 요인**

인지적 요인은 건강생성이론에서는 인지적 요인으로서 지식을 중대한 대처 자원 중의 하나로 언급하고 있다. 지식은 지식, 지능과 같은 것으로서, 지식은 통찰력을 주고 어떤 선택 상황에서 효과적 선택을 하도록 한다고 보았다(Antonovsky, 1985). 따라서 모야모야병 청소년의 질병 개념과 치료, 생활 관리 등 질병 관련 지식으로, 모야모야병의 관리를 위해 반드시 알아야 할 지식 관련 알아야 할 내용을 포함하여 구성하고자 한다.

**나. 정서적 요인**

정신, 심리적 자원의 요소로 긍정주의나 유머감각, 자기 존중감, 정서적 친밀감과 같은 것으로 모야모야병 청소년이 스트레스 상황에서 긍정적 방어기제 효과를 기대할 수 있다. 개인이 경험되어질 수 있는 상황에서 반응할 수 있는 긍정적 방어 혹은 해소 방법 등에 대해 공감할 수 있는 내용으로 구성하고자 한다.

**다. 사회적 자원**

사회적 자원은 구체적으로 인간관계적, 사회, 문화적 요소로 사회적 지지나 사회적 네트워크를 통해 관계된 자원이다. 다른 사람들과 친밀한 관계를 맺고 있는 사람은 그렇지 못한 사람들 보다 더 긴장을 쉽게 해소하며, 개인이 속한 환경이 건강향상과 관련이 있다. 따라서 모야모야병 청소년이 개인이 처한 상황에 따라 도움을 요청하고 돌봄을 받을 수 있으며, 자신이 속한 사회적 환경에서 가족, 학교에서 선생님, 친구들, 이용 가능한 의료 자원에 대해 공유하는 내용으로 구성하고자 한다.

**(2) 통합력**

통합력은 “역동적이고 지속적인 확신을 가진 총체적인 사고의 방향(global orientation)”으로 개인의 전체 상황을 이해하는 것과 이용 가능한 자원을 사용할 수 있는 능력으로 자신이 처한 상황을 평가하고 이해하고, 자원을 적절히 사용하여 건강 증진 방향으로 나아갈 수 있는 의미를 찾는 이해력, 관리력, 의미부여의 조합을 의미한다(Antonovsky, 1987). 본 연구에서는 통합력을 이해력, 관리력, 의미부여 구성 요소를 의미한다.

1. **이해력**

인지적 요인(cognitive component)으로 이해력(comprehensive)은 개인의 삶의 과정에서 내적과 외적 환경으로부터 오는 자극을 구조화하고, 예측하며, 설명할 수 있는 능력을 말한다(Antonovsky, 1987). 이에 모야모야병 청소년이 청소년 시기에서 정상적으로 겪게 되는 성장 발달적 경험과 더불어 자신의 질병과 관련된 신체적, 정서적 경험되어질 수 있는 모든 상황을 예측하며, 일관되게 쳬계화하는 능력을 증진시키기 위한 내용 구성이 필요하다. 즉, 모야모야병과 관련하여 개인에게 경험되어질 수 있는 내, 외부적 자극에 대해 예측하고, 경험되어진 상황을 개인이 설명할 수 있는 능력을 키울 수 있는 내용을 포함한다. 또한 청소년기 발달 단계에 따라 요구되어지는 도전과 미래준비와 관련한 내용을 포함하여 예측되는 스트레스 요인으로부터 긴장을 준비함을 통해 건강의 안락함의 삶의 방향으로 이끌고자 함이다.

**나. 관리력**

도구적, 구체적, 행동적 요인으로 관리력은 개인의 자원을 활용하거나 삶의 스트레스 요인으로 부터의 능동적 대처를 할 수 있는 가용한 내, 외적 자원을 활용할 수 있는 것이다(Antonovsky, 1987). 여기서 개인이 가용하다는 것은 자원이 자신의 통제하에 있다는 것은 물론 자신의 주위에 있는 부모님, 친구들, 의료진에 의해 통제할 수 있는 것도 포함한다. 따라서 모야모야병 청소년 자신들이 삶의 강에서 스트레스 요인에 직면했을 때, 활용 가능한 자원을 즉흥적, 장기적 계획에 따라 조정하는 훈련이 필요하다. 구체적 관리력 구성요인 내용은 긍정적인 해결의 대응책을 스스로 찾아가며, 자신의 상황을 향상시킬 수 있는 자기 조정을 통해 자신을 변화시기키는 내용의 중재 내용으로 구성하고자 한다.

**다. 의미부여**

동기적 요소(motivational component)로 의미부여는 개인의 삶은 의미가 있으며, 삶의 강에서 겪게 되는 어려움이나 문제들은 부담이 아닌 도전으로 여기며, 개인의 삶에 가치가 있는 것으로 여기고 참여를 하는 것을 의미한다(Antonovsky, 1987). 모야모야병 청소년들로 하여금 삶에 대한 의미부여는 자신의 존재 목적과 성취감을 느끼게 해주며, 가치있는 목적을 추구하고 있다는 믿음을 가지게 함으로써 살아갈 가치가 있다고 믿게 하는 것이 필요하다(Recker et al., 1987). 띠라서 구체적으로 자신에 대한 강점, 꿈, 인생에 대해 가치를 인식하여 어려움을 해결하려는 분명한 열망을 갖게 할 수 있는 내용과 때론 고통을 유발할 수 있는 스트레스 경험을 통해 에너지를 투자하려는 의지를 갖게 할 수 있는 내용으로 구성하고자 한다.

| **개 념** | **구성 요인** | | **구체적 구성 요소** | **방 법** | **전략** | **프로그램 원리** | **기대효과** |
| --- | --- | --- | --- | --- | --- | --- | --- |
| *일반적 저항자원 | 인지적 | | 질병관련지식 | 그룹 교육  개별 상담 | 질병관련 정보 제공,  전문적이며 구체적 접근 | 건강증진을 위한 요소, 대처 자원을 확장, 현재 자신과 관련된 일반적 저항자원과 잠재적 자원을 인식 증가 | 질병지식 향상, 개인 고유의 특별한 경험을 긍정적인 자원으로 활용, 지식은 통찰력을 제공. |
|  | 정서적 | | 긍정적인 상호작용(가족, 질병 그룹) | 그룹 토의 | 적극적인 경청, 피드백, 충고 |  | 친밀한 그룹관계 맺기,  가족의 지지체계를 이해, 가족과의 긍정적 상호작용 |
|  | 사회적 지원 | 인간관계적 | 사회적 지지 | 그룹 토의 | 사회적 지지 유용성 공유 |  | 사회적 지지 통한 중요한 대처 자원 |
|  |  | 사회·문화적 | 사회문화적 특성(학교, 교사, 문화적 요소) | 그룹 토의 | 친구, 교사, 학교생활에서의 지지, 전문의료지지에 대한 개인경험 공유 |  | 친구와 교사, 학교 생활, 전문의료지지에서의 상호작용 자원을 활용 |
| **통합력 | 이해력 | | 인지적 요소 | 개별 상담∙교육 | 탐색적 질문, 인지적 접근 | 능동적인 적응으로 개인의 독특한 역량 개발 | 내·외적 스트레스 자극에 대한 예측, 설명 |
|  | 관리력 | | 행동적, 도구적 요소 | 개별 상담∙ 교육 | 개별 중심적 상담을 통한 행동적 접근 |  | 내·외적 스트레스 자극에 대한 대처에 필요한 자원을 활용 |
|  | 의미부여 | | 동기적 또는 정서적 요소 | 개별 상담∙교육 | 개별적 접근, 수용, 공감적 이해 |  | 내·외적 스트레스 자극에 대한 도전, 집중 투자의 가치를 긍정적으로 받아들임. |

<표 1> 모야모야병 청소년의 건강생성 프로그램 구성요인 및 운영 방법(안)

**2) 구체적 프로그램 적용 방법**

프로그램 적용 방법은 그룹 접근 방법과 개별 접근 방법으로 구성하고자 한다. 그룹별 접근방법은 그룹 교육과 그룹 토의를 포함하며, 개별 접근방법은 개인 교육과 상담을 포함한다.

그룹별 접근방법(그룹 교육, 그룹 토의)은 모든 참여자에게 프로그램이 동일하게 적용되며, 일반적 저항자원의 구체적 내용을 구성하여 적용한다<표 3>

개별적 접근방법(개별 교육, 상담,)은 참여자 개인의 독특한 역량 개발을 목표에 따라 주제와 구체적 내용을 적용한다<표 2>

1. **그룹 교육**

그룹 교육은 연구자에 의해서 총 1회 제공하고자 한다. 참여자가 약 3-5명을 그룹화하여 총 6개의 그룹을 구성하며, 그룹 교육은 건강증진을 위한 일반적 저항자원과 잠재적 자원에 대한 인식을 높이고, 대처 자원을 확장하기 위함이다. 이를 위한 구체적 내용은 일반적 저항자원의 일차적 대인관계 수준레벨과 사회적 수준레벨에서의 일반적 저항자원에 대해 소개하기로 구성된다.

1. **그룹 토의**

그룹 토의는 연구자에 의해서 총 2회 제공하고자 한다. 참여자가 약 3-5명을 그룹화하여 총 6개의 그룹을 구성하며, 그룹의 상호작용 효과를 최대한 끌어내는 진행으로 각 회자 별 주제와 구체적 내용은 다음과 같다.

1회차 그룹 토의 주제는 나의 일반적 자원 및 잠재적 자원을 나누기 및 자신감 높이기이다. 이를 위한 구체적 내용은, 그룹 내에서 자신의 자원에 대해 이야기 하고, 다른 참여자의 자원에 대해 이야기를 들게 되면서, 자신이 깨닫지 못했던 자원에 대해서 알게 되며, 그룹 내 참여자들과 관계를 맺기를 시작하고, 다른 참여자에게 자신이 생각하는 자원에 대해 이야기를 하며, 진행자에 의한 적절한 피드백을 통해 긍정적 지지를 주는 것으로 구성하고자 한다.

2회차 그룹 토의 주제는 자원 탐색 및 대처 전략 세우기로 참여자 개인의 저항자원의 경험담과 기대되는 기능 및 이익에 대해 이야기를 나누고, 이를 바탕으로 미래의 대처 계획에 대해 발표하는 시간을 갖고, 진행자의 인도에 따라 서로의 계획에 추가 공유 의견을 나누는 것으로 구성하고자 한다.

1. **개별상담과 교육**

개인 상담 및 개인 교육은 연구자에 의해서 총 4회(1회/주) 제공된다. 참여자 개인의 일반적 저항자원의 개인레벨수준 구성요소와 통합력의 구체적 구성요소의 내용에 근거하여 개인의 독특한 경험적 특성을 통한 대처능력을 향상시키고, 개인의 일반적 저항 자원을 인식하고 구체적으로 관리할 수 있는 행동과 자신의 삶의 의미를 부여를 통해 건강증진의 동기 부여를 이끌 수 있는 내용으로 구성된다.

**3) 프로그램 회기 수 및 회기 시간 결정**

건강생성이론 원칙에 근거한 중재 프로그램 연구에서 참여자들의 건강생성 요소를 높이기 위해 긍정적인 변화를 가져올 수 있는 어느 정도의 회기와 시간으로 구성되어야 한다는 기준이 없기 때문에(Lim, 2011) 기존 청소년 대상 선행되어진 연구를 기준으로 살펴본 결과 6-12회기까지 다양하였고, 집단 혹은 그룹 교육의 경우 60분 정도로 본 연구에서는 희귀 난치성 질환 청소년 그룹을 감안하여 집단 교육을 1회, 집단 토의를 2회, 개별 상담 및 토의는 총 4회, 교육 시간은 60분으로 운영할 예정이다. 희귀 난치성 질병군인 것과 학교생활을 하고 있는 청소년기를 고려하여 한 그룹은 총 3-5명정도로 구성하여 6개 집단을 운영할 계획이다. 따라서 참여자 개인에게 운영되어지는 횟수는 총 7회로 구성할 예정이다. 그러나 향후 개별 심층 면담과 포커스 그룹 인터뷰 결과를 분석하여 최종 수정할 예정이다.

<표 2>. 교육 회기별 프로그램의 구성요소 및 내용(안)

| 회차 | 방법 | 개념 | 구성요소 | 중재내용 | 소요시간 |
| --- | --- | --- | --- | --- | --- |
| 1차 | 그룹교육 | GRR* | 인지적 요소 | ∙ 모야모야 병 개념과 치료  ∙ 모야모야 병 환자의 생활  ∙ 뇌 관리, 아는 것이 힘이다  ∙ 기타 관리 지침(건강행위의 중요성) | 70분 |
| 2차 | 그룹토의Ⅰ | GRR* | 정서적 요소 | ∙현실적 상황에 대해서 이야기 나누고 공감, 피드백  ∙친밀한 또래관계 맺기: 그룹 상호작용 | 70분 |
| 3차 | 그룹토의Ⅱ | GRR* | 인간관계적, 사회문화적 요소 | ∙인간관계/사회 문화적 자원에 대해 이야기 나누고 피드백  ∙이용 가능한 의료 자원에 대해 공유  ∙자신의 환경에서의 지지 체계에 대해 스스로 확인, 발표, 그룹 전체 공감 | 70분 |
| 4차 | 개별상담Ⅰ | SOC** | 이해력 | ∙과거 내, 외적 자극에 발생하였던 사례 들을 이야기  ∙청소년 개인의 역동적인 일상생활에서 발생할 수 있는 상황 혹은 자극을 스스로 예측  ∙개인의 생활에서의 예측되는 자극이 일어나는 이유를 스스로 설명  ∙이해되어지는 상황들을 기록 | 40분 |
| 5차 | 개별상담Ⅱ | SOC** | 관리력 | ∙과거 사례에서 스트레스 상황에 어떤 자원을 적절히 사용했는지 회상  ∙예측 가능한 스트레스 상황에 필요한 활용 가능한 자원을 스스로 기록 | 40분 |
| 6차 | 개별상담Ⅲ | SOC** | 의미부여 | ∙ 과거 내, 외적 스트레스 자극이 자신에게 어떤 의미가 있었는지 회상  ∙ 예측 가능한 스트레스 자극 상황이 개인의 삶에 어떤 도전을 주고 집중하기 위해 투자의 가치가 있는지 스스로 기록 | 40분 |
| 7차 | 개별상담Ⅳ | SOC** | 이해력, 관리력, 의무부여 | ∙ 4,5,6차때 기록한 내용을 바탕으로 통합적 연결을 통한 자신감을 확인  ∙ 건강생성에서의 건강의 의미를 재확인  ∙ 통합력을 향상시키고자 하는 개인의 의지에 대한 칭찬과 긍정적 피드백 제공 | 40분 |

*GRR(Generalized Resistence Resources):일반적 저항자원,

**SOC(Sense of Coherence):통합력

**2단계 : 모야모야병 청소년의 건강생성 프로그램 효과 검증**

1. **연구 설계**

본 연구는 모야모야병 청소년의 건강증진을 위한 건강생성 프로그램을 적용하고 그 효과를 확인하기 위한 무작위 배정 대조군 전후설계의 실험연구이다.

본 연구의 설계를 도식화 하면 다음과 같다.

|  | 사전조사 | 실험처치 | 사후조사 | |
| --- | --- | --- | --- | --- |
| 중재군 | E1 | X1 | | E2 |
| 비교군 | C1 | Y1 | | C2 |

E1, C1 : 질병관련 지식, 인지된 사회적 지지, 통합력, 청소년 스트레스, 모야모야병 청소년의 건강행위, 우울, 허혈 발생빈도, 주관적 건강상태, 삶의 질

X1 : 실험처치; 모야모야병 청소년의 건강증진을 위한 건강생성 프로그램

Y1 : 대조군 교육 ; 일대일 1회 교육으로 현재 임상에서 진행되어지고 있는 질환, 수술, 전후 주의사항

E2, C2 : 질병관련 지식, 인지된 사회적 지지, 통합력, 청소년 스트레스, 모야모야병 청소년의 건강행위, 우울, 허혈 발생빈도, 주관적 건강상태, 삶의 질

1. **연구대상자 및 실험 배정**

**구체적 연구 대상자 선정 및 실험 배정 법은 p.5 연구 대상자 선정 내용 참고**

본 연구는 모야모야병 청소년의 건강증진을 위한 모야모야병 건강생성 프로그램을 개발하는 연구(1단계)와 개발된 모야모야병 건강생성 프로그램의 효과를 검정하기 위한 실험연구(2단계)로 구성할 예정이다.

1. **자료 수집 및 절차**

본 연구의 자료수집 기간은 2018년 00월 00일부터 00월 00일까지 예정이며 자료수집 방법은 모야모야병 청소년과 부모 혹은 법정 대리인에게 본 연구의 목적을 설명한 후 참여에 동의한 경우 대상자의 시간을 고려하여 미리 자료수집 시간을 정할 예정이며, 직접 만남을 통해 자료 수집을 할 예정이다.

1. **사전조사**

모집할 대상자에게 설문조사와 일반적 질병관련 병력을 조사할 예정이다. 설문지 작성은 15분 정도 소요될 예정이다.

1. **실험적용**

본 연구의 비교군은 모야모야병 진단받은 청소년으로 일반적으로 현재 병원에서 시행되고 있는 일대 일 교육만 제공되어질 것이며, 중재군은 모야모야병 청소년 건강생성 프로그램이 적용되어질 것이다. 현재 진행되어지고 있는 일대일 교육은 모야모야 질병에 대한 소개, 일상생활 주의 사항, 수술 전, 후 교육, 퇴원 후 관리로 입원 시 1회 병실에서 약 10분간의 일대 일 교육을 말한다.

1. **사후조사**

사후조사는 중재군의 경우 모든 프로그램이 종료된 시점으로 사전조사에서 조사되어진 모든 변수와 프로그램 만족도에 대한 개인 의견을 조사할 예정이다. 비교군의 경우 현재 시행되어지고 있는 일대 일 교육 전에 시행하며, 모든 프로그램 종료 후에 시행할 예정이다.

실험 도중 초래될 수 있는 내적 타당도의 위험요소를 제거하기 위해 실험 상황을 다음과 같이 통제할 예정이다.

첫째, 측정도구상의 문제를 최소화하고 내적 타당도를 높이기 위해, 사전, 사후 측정 모두 본 연구자가 직접 자료 수집할 예정이다.

둘째, 제 3변수의 개입을 막기 위해 실험처치 기간 동안 다른 기관에서 시행되는 건강강좌나 교육 등에 참여하지 않도록 연구 대상자에게 교육할 예정이다.

본 연구의 구체적인 자료수집 진행절차(안)는 다음과 같다<그림 1>

**사전 조사**

**실험처치**

(7주간)

**사후 조사**

입원일 또는 외래방문일

실험군 : 프로그램 적용 직 후

대조군 : 사전 조사 후 7주

자료수집일정

실 험 군

- 인구학적 특성
- 질병관련 특성
- 질병관련 지식
- 인지된 사회적 지지
- 통합력
- 청소년 스트레스
- 모야모야병 청소년의 건강행위
- 우울
- 허혈 발생빈도
- 주관적 건강상태
- 삶의 질
- 질병관련 지식
- 인지된 사회적 지지
- 통합력
- 청소년 스트레스
- 모야모야병 청소년의 건강행위
- 우울
- 허혈 발생빈도
- 주관적 건강상태
- 삶의 질

건강생성 프로그램(7주간)

대 조 군

- 인구학적 특성
- 질병관련 특성
- 질병관련 지식
- 인지된 사회적 지지
- 통합력
- 청소년 스트레스
- 모야모야병 청소년의 건강행위
- 우울
- 허혈 발생빈도
- 주관적 건강상태
- 삶의 질
- 질병관련 지식
- 인지된 사회적 지지
- 통합력
- 청소년 스트레스
- 모야모야병 청소년의 건강행위
- 우울
- 허혈 발생빈도
- 주관적 건강상태
- 삶의 질

일대 일 교육 1회

<

<그림 1> 본 연구의 자료수집 진행절차(안)

**13. 관찰 항목**

위의 그림 1과 같이 인구학적 특성 , 질병관련 특성, 질병관련 지식 , 인지된 사회적 지지

, 통합력, 청소년 스트레스, 모야모야병 청소년의 건강행위, 우울, 허혈 발생빈도, 주관적 건강상태,삶의 질을 측정할 예정이다.

**1)연구의 도구**

1. **인구학적 특성**

본 연구의 인구학적 특성 조사를 위해 성별, 나이, 거주 가족, 출석학교, 흡연 및 음주 여부 등의 일반적 특성의 조사를 위해 양자택일, 선다형 질문지 형식으로 구성하여 측정할 것이다. ,

1. **질병관련 특성**

본 연구의 질병관련 특성으로는 Suzuki stage 에 따른 분류에 해당하는 병기, 질병을 진단 받은 기간, 뇌출혈이나 뇌경색의 진단여부, 경련 경험 유무와 횟수, 일과성 허혈성 발작(TIA)의 유무와 횟수, 모야모야병 관련 수술 여부 및 종류, 현재 복용중인 약물 여부, 동반하고 있는 질환의 유무 등으로 의무기록자료(EMR)를 통해 조사할 예정이다.

1. **질병 관련 지식**

본 연구자가 모야모야병과 관련된 중요한 질병 관련 지식 문항을 만든 후 전문가 타당도를 거쳐, 모야모야병 청소년 아이들에게 pilot test를 통해 문항의 이해도 등을 미리 확인할 예정이다. 측정하는 각 문항에 대해 옳게 응답한 경우 1점을, 모른다 또는 틀린 경우 0점을 부여할 것이고, 측정된 점수가 높을수록 모야모야병 관련 지식이 높음을 의미한다. 본 도구의 신뢰도는 KR-20을 통하여 측정할 것이다.

1. **인지된 사회적 지지**

인지된 사회적 지지란 사회적 관계에서 사람들의 일반적 지원 또는 특정 지원에 대한 개인의 인식으로 Malecki 과 Demaray (2000)이 개발한 아동 청소년 대상의 사회적 지지 도구로 교사, 부모, 친한 친구, 학급친구)별로 사회적 지지를 나누는 4 개의 하부 조직으로 나뉘며, 총 60문항이나 한국적 문화에 맞게 본 연구에서는 교사, 부모, 친한 친구 하부 항목인 36개 항목만을 사용할 예정이다. 빈도 등급은 6 점의 리 커트 척도로 1 (Never)에서 6 (Always)까지 이며, 각 문항당. 중요도 등급은 1 점 (중요하지 않음)에서 3 점 (매우 중요 함)까지의 3 점 리 커트 척도로 구성된다. 도구 개발 당시 신뢰도는 Cronbach’s α=.96이었다.

본 도구는 CASSS의 저작권을 가지고 있는 Malecki 과 Demaray (2000)으로부터 도구사용허가를 받았고, 이 도구는 번역, 역번역 과정을 거친 것을 사용할 예정이다.

1. **통합력**

통합력은 일상생활에서 불가피한 스트레스를 다루기 위한 전체적인 상황을 이해하는 능력과 자원을 잘 활용하는 능력의 합을 말하며(Antonovsky, 1987), Antonovsky(1987)가 개발한 13문항의 통합력 측정 도구를 연구자가 번역-역번역을 거쳐 번안하여 모야모야병 청소년군을 대상으로 도구의 타당도를 확인한 후 사용예정이다. 도구의 개발당시 신뢰도는 Cronbach’s α는 0.86 이었고(Antonovsky, 1993), Unni et al(2016)의 연구 보고에서는 Cronbach’s α는 0.92로 나타났다.

통합력 도구는 Antonovsky(1987)에 의해 2가지 도구가 개발되었으며, Eriksson & Lindstrom(2005)의 Antonovsky(1987)의 Sense of Coherence Scale에 대한 체계적 분석 연구에서 통합력 29문항 도구는 Cronbach’s α 는 0.70 ~ 0.95, 통합력 13문항 도구는 Cronbach’s α 값이 0.70 ~ 0.92로 보고한 바 있다.

본 연구에서는 통합력 13문항의 도구를 사용할 예정으로 도구는 통합력의 저작권을 가지고 있는 Sweden Health & Culture University West의 Monica Eriksson으로부터 도구 사용 허가를 받고, 한국어로 번역, 역 번역의 과정을 거친 것을 사용 할 예정이다.

1. **스트레스**

스트레스를 측정하기 위해 김병선과 배성만(2014)이 우리나라 청소년의 실정에 맞게 개발한 청소년용 일상적 스트레스반응 척도로 스트레스 척도의 하위요인의 문항구성은 신체적 스트레스, 행동적 스트레스, 정서적 스트레스 총 27문항으로 구성되었다. 본 척도는 4단계 Likert식 척도로 ‘전혀 그렇지 않다(1)’, ‘별로 그렇지 않다(2)’, ‘조금 그렇다(3)’, ‘매우 그렇다(4)’의 응답범주로 4점으로구성되었다. 전체 문항 수는 27문항으로 각 하위요인의 점수가 높을수록 해당되는 요인의 스트레스를 많이 경험하는 것을 의미한다. 도구의 개발당시 신뢰도는 Cronbach’s α는 0.82 이었다.

1. **모야모야병 건강행위**

모야모야병 건강행위란 모야모야병을 경험하는 청소년이 인지하는 건강행위와 건강을 유지하기 위해 필요한 건강행위, 증상 발현시 해야 하는 행위, 청소년 시기에 건강 위협 행위 등을 포함하는 건강행위를 측정하기 위해 Yeom and Oh(2018)가 청소년 대상 건강행위 관련 문헌고찰과 모야모야병 관련 건강행위를 전문가 그룹 인터뷰를 통해 문항을 추출 한 후 모야모야병 청소년 대상으로 타당도 검증을 거쳐 도구를 개발하였다. 구체적 하위요인으로는 생활습관, 건강 대처, 정신 건강, 치료 이행으로 총 24문항으로 5단계 Likert 척도로 점수가 높을수록 해당하는 건강행위 이행이 높은 것을 의미한다. 도구의 개발 당시 신뢰도는 Cronbach’s α는 0.80 이었다.

1. **우 울**

청소년의 우울 정도를 평가하기 위해 Kovacs(Kovacs, 1985; Kovacs, 2003)가 개발한 CDI(Children‘s Depression Inventory)를 조수철 등이 번안한 척도(Cho & Lee, 1990)를 본 연구에 사용 예정이다. 검사는 우울정서, 행동장애, 흥미상실, 자기비하, 생리적 증상의 5가지 하위 범주로 한 총 27문항으로 이루어져있다. 검사는 증상의 심각도를 0점에서 2점까지 평정하며 점수의 범위는 0점에서 54점이다. 조수철 등이 시행한 연구(Cho & Lee, 1990)에서 CDI의 내적 일치도는 .88로 나타났다. 또한 김은경 등 이 시행한 연구(Kim et al., 2005)에서 청소년에 대한 CDI 의 내적 일치도가 .87로 나타나 본 연구의 연구 대상인 청소년에서 CDI를 적용할 예정이다.

**⑨ 주관적 건강상태**

주관적 건강상태는 모야모야병 청소년이 현재 자신의 건강상태에 대한 지각하는 건강에 대한 주관적 평가로 5점 척도 리커트 척도로 1점(아주 나쁘다), 5점(아주 좋다)까지로 구성한 본 연구를 위해 연구자가 개발된 단순 척도이다.

**⑩ 허혈 발생빈도**

모야모야병 증상으로 마비나 감각이상, 언어장애, 시력장애 등의 개인의 주 증상을 하루에 나타난 평균 빈도로 직접 표시하는 횟수를 의미한다.

**⑪ 삶의 질**

삶의 질 정도는 Varni, Seid와 Kurtin (2001)이 개발한 소아용 삶의 질 검사 제4판(Pediatric Quality of Life InventoryTM4.0 Generic Core Scales)을 Kook과 Vanri (2008)에 의해 번역, 표준화 과정을 거친 한국 어 소아용 삶의 질 검사 제4판(The Korean Translation of the Pediatric Quality of Life InventoryTM4.0 Generic Core Scales)을 사용하여 측정할 것이다. 지각하는 삶의 질을 측정하는 PedsQLTM 4.0은 만 8세에서 12세 를 위한 아동용과 만 13세에서 만 18세를 위한 청소년용 두 종류이며 모두 동일한 문항으로 아동 보고용과 부모 보고용으로 구성되어, 아동 자신이 지각하는 삶의 질 수준과 부모가 지각하는 아동 삶의 질을 평가할 수 있다. 본 연구에서 만 13세에서 만 18세 청소년용 도구만을 사용할 예정이다. 본 도구는 신체적 삶의 8문항, 정서 기능 5문항, 대인관계 기능 5문항, 학교 기능 5문항의 총 23문항으로 구성되어있으며 각 문항은 ‘전혀 없다(0점)’, ‘거의 없다(1점)’, ‘가끔 있다(2점)’, ‘자주 있다(3 점)’, ‘매우 자주 있다(4점)’ 5점 척도 이며, 채점은 0을 100점으로, 1점 을 75점으로, 2점을 50점으로, 3점을 25점으로, 4점을 0점으로 환산하여 합한 총점을 총 문항 수로 나눈 평균 점수를 사용하며, 점수가 높을 수록 삶의 질이 높음을 의미한다. 표준화 연구에서 아동 보고용 도 구의 전체 문항 신뢰도는 .90 이었다. Choi, Kim, Chung, Park과 Lee (2010)의 연구 보고에서는 아동 보고용 도구의 전 체 문항에 대한 신뢰도는 .94로 나타났다.

**14. 효과 평가 기준 및 방법**

연구의 효과를 확인하는 방법은 관찰 항목 중에서 프로그램 적용을 통해 질병관련 지식, 인지된 사회적 지지, 통합력, 청소년 스트레스, 모야모야병 청소년의 건강행위, 주관적 건강상태, 삶의 질은 높아질 것이라는 것과 반면 우울, 허혈 발생빈도는 낮아질 것을 기대하고 있다.

**15. 자료분석과 통계적 방법**

수집된 자료는 SPSS WIN(20.0)을 이용하여 분석할 예정으로 구체적인 방법은 다음과 같다.

1. 실험군과 대조군의 일반적 특성, 제 연구변수의 분포 특성은 실수, 백분율, 평균, 표준편차 등의 기술통계로 산출할 예정이다.
2. 일반적 특성 및 종속 변수에 대한 정규성 검정은 Shapiro-Wilk를 이용할 예정이다.
3. 실험군과 대조군의 일반적 특성과 사전 동질성 검증은 X²-test와 t-test를 이용하여 분석할 예정이다.
4. 가설검증은 t-test, Paired t-test를 이용하여 분석할 예정이다.

**16. 중지 및 탈락기준**

연구의 목표 대상자가 모집되면, 자료 수집은 종료하고 연구 분석 방법을 통해 연구를 중지할 예정이다. 조기중단의 기준은 연구 대상자를 모집하기 어려운 경우 연구를 조기 중단할 예정이다. 또한 대상자가 연구에 지속적 참여가 어려운 건강상태나 연구 진행에 영향을 주게 되는 상황이 발생할 시 연구를 중지할 예정이다.

추가 구체적 예는 다음과 같다.

1. 대상자가 연구 참여동의를 철회한 경우(피험자가 연구 중지/탈락을 요구하는 경우 어느 시점에서든 연구 중지/탈락 가능)
2. 질병 특성상 증상 악화 등으로 연구 참여가 어렵거나 갑작스러운 사고로 인하여 연구참여가 어렵다고 판단한 경우.

**17. 연구대상자의 위험과 이익**

연구에 있어서 대상자에게 예상되는 위험성은 침습적이거나 치료에 영향을 주는 항목이 없으므로 현재 없는 상태이며, 심층면담 혹은 설문지를 하는 동안 개인적 시간을 할애해야하는 참여가 예상되나 연구에 대한 설명과정에서 이 문제까지 동의한 경우에 참여시킬 예정이다. 2단계 연구에서 중재군의 해당하는 경우 프로그램의 기대 효과를 경험할 수 있다는 이익을 기대할 수 있다.

**18. 연구대상자 안전대책 및 개인정보보호대책**

**연구 대상자 안전대책**

대상자 안전과 연구자료의 완결성을 보증하기 위해 연구 책임자는 1달에 한번 연구팀 1명이상과 동의서 확인, 대상자 안전성 검토, 연구의 유효성을 검토하기 위해 중간분석 실시할 예정이다.

**개인정보보호 및 연구자료의 기밀 유지를 위한 방안**

연구 대상자를 보호하기 위해서 연구 참여로부터 얻은 자료 중 모든 개인정보는 삭제하고 식별정보에 코드를 부여하여 노출을 최소화할 예정이다. 면담 필사본, 설문지와 연구 결과는 연구 이외의 목적으로는 이용되지 않을 것이며 연구자료는 엄격하게 대상자의 비밀이 유지되어 보호받게 될 것이다. 대상자가 제공한 연구 자료는 대상자 본인이 요청하지 않는 한 보호자를 포함한 누구에게도 공개하지 않도록 하며 이와 같은 자료 수집 전 대상자가 충분히 인지할 수 있도록 설명을 제공하고 동의서를 받을 것이다. 자료의 기밀성을 위해서 연구자료는 주 연구자가 보관하고 보관서랍에는 잠금 장치를 설정하여 관리하고, 면담자료의 기록 파일은 비밀번호를 설정하여 관리할 예정이다. 수집된 자료는 이중 잠금 장치 된 별도의 보관장소에 둘 예정임. 연구가 종료되면 수집된 자료들은 밀봉 박스 처리하여 3년이 경과 후에 폐기 처분할 예정이다.

**19. 참고문헌**

1. 김덕경, 장성아, 박택규(2016). 모야모야병: 순환기 의사가 보는 관점. *한국 지방 지질학회 및 동맥 경화 학회지*, 5(2), 115-120.
2. 김병선, 배성만 (2014). 청소년용 일상적 스트레스 반응 척도 개발과 타당화 연구, *한국심리학회지*, 11(1), 19-32.
3. 김정호 & 김선주(2007). 스트레스의 이해와 관리. *서울: 시그마프레스*.
4. 김창대, 김형수, 신을진, 이상희, 최한나(2011). 상담 및 심리 교육 프로그램 개발과 평가. *서울: 학지사*.
5. 고기홍 (2003). *청소년 스트레스 관리 프로그램 개발에 관한 연구*. 동아대학교 대학원 박사 학위논문.
6. 양승연 (2004). *청소년의 자살생각과 스트레스와의 관계에 관한 연구*. 한남대학교. 석사학위논문.
7. 이미자, 김민주 (2016). 만성질환자의 건강행위 실천을 위한 의지(volition)에 대한 개념분석*. J Korean Acad Nurs* ,46(5),687-696.
8. 성기숙(2005). *만성질환 아동의 극복력 증진을 위한 집단치료놀이 프로그램의 개발 및 효과*, 고려대학교, 박사논문
9. 조수철, 이영식(1990). 한국형 소아우울척도의 개발. *신경정신의학회지*, 29(4), 943-955.
10. 최창용(2015). 아토피 피부염 청소년의 삶의 만족도에 영향을 미치는 요인. *한국청소년연구*, 26(1),, 111-144.

# [**Anna, Z.**](https://www.ncbi.nlm.nih.gov/pubmed/?term=Zashikhina A%5BAuthor%5D&cauthor=true&cauthor_uid=24460738) and [**Bruno, H.**](https://www.ncbi.nlm.nih.gov/pubmed/?term=Hagglof B%5BAuthor%5D&cauthor=true&cauthor_uid=24460738) (2014). Health-related quality of life in adolescents with chronic physical illness in northern Russia: a cross-sectional study*.* [***Health Qual Life Outcomes***](https://www.ncbi.nlm.nih.gov/pmc/articles/PMC3905674/). 12(12).

1. Antonovsky, A. (1979). Health, Stress and Coping. *San Francisco*: Jossey-Bass*.*
2. Antonovsky, A. (1987). Unraveling the mystery of health: How people manage stress and stay well. *San Francisco*: Jossey-Bass.
3. Antonovsky, A. (1993). The salutogenic approach to aging. *Lecture held in Berkeley*, 2(21).
4. Antonovsky, A. (1996). The salutogenic model as a theory to guide health promotion*.. Health Promotion International*, 11(1), 11-18.
5. Aldwin, C. M., & Revension, T. A.(1987). Does coping heip? A reetamination of the relation between coping and mental health. *Journal of Personality and Social Psychology*, 53, 337-348.
6. Apers, S., Moons, P., Goossens, E., Luyckx, K., Gewillig, M., Bogaerts, K. (2013). Sense of coherence and perceived physical health explain the better quality of life in adolescents with congenital heart disease. European Journal of Cardiovascular Nursing, 12(5), 475–483.
7. Ayo, Y. O., Reddy, P. S., & Van, B. W. (2009). Longitudinal association of adolescents‘ sense of coherence with tooth‐brushing using an integrated behavior change model. *Community Dentistry and Oral Epidemiology*, 37(1), 68–77.
8. Bang, O. Y., Fujimura, M., & Kimc, S. K. (2016). The Pathophysiology of Moyamoya Disease. *An Update. Journal of Stroke*, 18(1), 12-20.
9. Barlow, J. H. & Ellard, D. R. (2006). The psychosocial well-being of children with chronic disease, their parents and siblings: An overview of the research evidence base. *Child: Care, Health and Development*, 32(1), 19-31.
10. Baker L.K. & Denyes M.J. (2008) Predictors of self-care in adolescents with cystic fibrosis: a test of Orem‘s theories of self-care and self-care deficit. Journal of Pediatric Nursing 23 (1), 37–48.
11. Bao, X. Y., Duan, L., Yang W. Z., Li, D. S., Sun, W. J., Zhang, Z. S., Zong, R., Han, C. (2015) Clinical features, surgical treatment, and long term outcome in pediatric patients with moyamoya disease in China. *Cerebrovasc Dis,* 39(2), 75–81.
12. Beck, A. T. (1972). Depression: Causes and Treatment*. Philadelphia*: University of Pennsylvania Press.
13. Bersano, A., Guey, S., Bedini, G., Nava, S., Hervé, D., Vajkoczy, P., Klijn, C. (2016). Research Progresses in Understanding the Pathophysiology of Moyamoya Disease. *Cerebrovascular Diseases*, 41(34), 105-118.
14. Bronikowski, M. & Bronikowska, M. (2009). Salutogenesis as a framework for improving health resources of adolescent boys. Scandinavian Journal of Public Health, 37*(*5), 525–531.
15. Bengtsson, T. A., & Hansson, L. (2001). The validity of Antonovsky’s sense of coherence measure in a sample of schizophrenic patients living in the community. *Journal of* *Advanced Nursing*, *33*(4), 432-438.
16. Braun, L. O. (2014). Coping resources and stress reactions among three cultural groups one year after a natural disaster. *Clinical Social Work Journal*, 42, 366–374.
17. Braun, L. O., Sagy, S., & Roth, G. (2010a). Coping strategies among adolescents: Israeli Jews and Arabs facing missile attacks. Anxiety Stress and Coping, 23(1), 35–51.
18. Blom, E. C. H., Serlachius, E., Larsson, J. O., Theorell, T., & Ingvar, M. (2010). Research low sense of coherence (SOC) is a mirror of general anxiety and persistent depressive symptoms in adolescent girls-a cross-sectional study of a clinical and a non-clinical cohort*. Health and Quality of Life Outcomes*, 8, 58.
19. Choi, B. Y., Kim, D. H., Chung, K. M., Park, M. J., & Lee, E. B. (2010). Psychosocial characteristics of girls with Turner syndrome and age-matched healthy control. *Korean Psychological Association Woman,* 15(3), 489-507.
20. Cho, S.C. and Lee, Y.S. 1990. Development of the Korean form of the Kovacs’ Childeren’s Depression Inventory. *Journal of Korean Neuropsychiatric Association,* 29(4): 943- 956.
21. Chmlin, S. L. & Chren, M. M. (2010). Quality of life outcomes and measurement in childhood atopic dermatitis. *Immunology and Allergy Clinics of North America*, 30(3), 281-288.
22. Delgado, C. (2007). Sense of coherence, spirituality, stress and quality of life in chronic illness. *Journal of Nursing Scholarship,* *39*(3), 229-234.
23. Dubow,E. F.,& Tisak,J.(1989).Therelation between stressfullifeevents and adjustmentin elementary schoolchildren:Theroleof social support and social problem-solving skills. *Child* *Development, 60,* 1412-1423.
24. Eriksson, M., & Lindström, B. (2005). Validity of Antonovsky’s sense of coherence scale: a systematic review. *Journal of* *Epidemiology and Community Health, 59*(6), 460-466.
25. Fujimura, M., Sonobe, S., Nishijima, Y., Niizuma, K., Sakata, H., Kure, S. (2014). Genetics and Biomarkers of Moyamoya Disease: Significance of RNF213 as a Susceptibility Gene, [*J Stroke*.](https://www.ncbi.nlm.nih.gov/pubmed/24949311) 16(2), 65-72.
26. Fok, S. K., Chair, S. Y., & Lopez, V. (2005). Sense of coherence, coping and quality of life following a critical illness. *Journal of Advanced Nursing, 49*(2), 173-181.
27. Garcı´a, M. I., Moreno, C., & Braun, L. O. (2013). Neighbourhood perceptions and sense of coherence in adolescence. The Journal of Primary Prevention, 34(5), 371–379.
28. .Garcı´a, M I., Moreno, C., & Jime´nez, I. A. (2013). Understanding the joint effects of family and other developmental contexts on the sense of coherence (SOC): A person-focused analysis using the classification tree. *Journal of Adolescence,* 36(5), 913–923.
29. Garcı´a, M. I., Rivera, F., & Moreno, C. (2013). School context and health in adolescence: The role of sense of coherence. *Scandinavian Journal of Psychology*, 54(3), 243–249.
30. Garnefski, N., Kraaij, V., & Spinhoven, P. (2001). Negative life events, cognitive emotion regulation and emotional problems. *Personality and Individual Differences,* 30(8), 1311–1327.
31. Gauffin, H., Landtblom, A. M., & Ra¨ty, L. (2010). Self-esteem and sense of coherence in young people with uncomplicated epilepsy: A 5-year follow-up. Epilepsy & Behavior, 17(4), 520–524.
32. Geckova, A. M., Tavel, P., van, D. J., Abel, T., & Reijneveld, S. (2010). Factors associated with educational aspirations among adolescents: Cues to counteract socioeconomic differences? BMC. *Public Health*, 10(1), 154.
33. Glanz, K., Gertraud, M., & Carlin, L. (2005). Ethnicity, sense of coherence, and tobacco use among adolescents. *Annals of Behavioral Medicine*, 29(3), 192–199.
34. Gochman, D. S.(1988). Health behavior **:** emerging research prospectives Plenum Press, *New York*.
35. Hampel, P., Rudolph, H., Stachow, R., & Petermann, F. (2003). Multimodal patient education program with stress management for childhood and adolescent asthma. Patient Education and Counseling, 49(1), 59–66.
36. Haoka, T., Sasahara, S., Tomotsune, Y., Yoshino, S., Maeno, T., & Matsuzaki, I. (2010). The effect of stress-related factors on mental health status among resident doctors in Japan. *Medical Education*, 44(8), 826-834.
37. Hatherill, S. (2007). Psychiatric aspects of chronic physical illness in adolescence：significant numbers of chronically ill adolescents have problems coping with their illness. *Continuing Medical Education*, 25(5), 212-214.
38. [Hoshino, H](http://www.ncbi.nlm.nih.gov/pubmed/?term=Hoshino H%5BAuthor%5D&cauthor=true&cauthor_uid=22688065)., [Izawa, Y](http://www.ncbi.nlm.nih.gov/pubmed/?term=Izawa Y%5BAuthor%5D&cauthor=true&cauthor_uid=22688065)., [Suzuki, N](http://www.ncbi.nlm.nih.gov/pubmed/?term=Suzuki N%5BAuthor%5D&cauthor=true&cauthor_uid=22688065). (2012). Research Committee on Moyamoya Disease. Epidemiological features of Moyamoya disease in Japan. [*Neurologia Medico-Chirurgica (Tokyo)*](http://www.ncbi.nlm.nih.gov/pubmed/22688065), *52*(5): 295-298.

# Hwang Y.S.(2010). Clinical Features of Moyamoya Disease: An Overview. [***Moyamoya Disease Update***](https://link.springer.com/book/10.1007/978-4-431-99703-0)**; 107-109.**

1. Ihm, M. O., Song, M. K., & Kim, C. S. (2012). A mediating model of social support between anger and psychological maladaptation of adolescents. *Korean Journal of Youth Studies*, 19(4), 247-270.

# Ishikawa, T., Tanaka, N., Houkin, K., Kuroda, S., Abe, H., Mitsumori, K. (1998). Regional cerebral blood flow in pediatric Moyamoya disease: Age-dependent decline in specific regions. *Child‘s Nervous System*, 14(8):366-71.

1. Kleinloog, R., Regli, L., Rinkel, G. J., Klijn, C. J. (2012). Regional differences in incidence and patient characteristics of moyamoya disease: a systematic review. *J Neurol Neurosurg Psychiatry*, 83(5), 31-6.
2. Koposov, R. A., Ruchkin, V. V., & Eisemann, M. (2003). Sense of coherence: A mediator between violence exposure and psychopathology in Russian juvenile delinquents. *The Journal of Nervous and Mental Disease*, 191(10), 638–644
3. Kovasc, M. (1985). The Children’s Depression, Inventory (CDI). *Psychopharmacology Bulletin* , 21(4): 995-998.
4. Kovasc, M. (2003). Children‘s depression inventory (CDI) Technical manual update. *Toronto, Canada*: Multi-Health Systems. Pp. 96.
5. Kook, S. H., & Varni, J. W. (2008). Validation of the Korean version of the pediatric quality of life inventory 4.0(PedsQL) generic core scales in school children and adolescents using the Rasch model. *Health and Quality of Life Outcomes,* 2(6), 41.
6. Kim J.S. (2016) Moyamoya disease: epidemiology, clinical features, and diagnosis. *J Stroke*, 18(1):2–11.
7. Kim, H. Y., Chung, C. S., Lee, J., Han, D. H., Lee, K. H. (2003). Hyperventilation-induced limb shaking TIA in Moyamoya disease. *Neurology*. 60(11), 137-9.
8. Kim, J. (2015). Mental Health in Adolescents with Allergic Diseases-Using Data from the 2014 Korean Youth’s Risk Behavior Web-based Study*. J Korean Soc Sch Health*. 28(2), 79-88.
9. Kim, Y.J. (2006). *The relationships among communication in parentchild, stress coping and adolescents’ school adjustment* [master’s thesis]. Seoul: Seoul Women’s University;. P. 1-80.
10. Lim, H. S. (2011). *The development and effectiveness of the family resilience enhancement program forthefamily of chronic schizophrenic patient*[dissertation].Seoul:
11. Li, X., Chi, P., Sherr, L., Cluver, L., Stanton, B. (2015). Psychological Resilience among Children Affected by Parental HIV/AIDS: A Conceptual Framework. *Health Psychol Behav Med*, 3(1), 217-235.
12. Malecki, C. K., Demaray, M. K., & Elliott, S. N. (2000). *The Child and Adolescent Social Support Scale*. DeKalb: Northern Illinois University.
13. Masten A. S.(2001). Resilience in children threatened by extreme adversity: Frameworks for research, practice, and translational synergy. Development and Psychopathology. 23(2),493–506.
14. Mattila, M. L., Rautava, P., Honkinen, P. L., Ojanlatva, A., Jaakkola, S., & Aromaa, Ml. (2011). Sense of coherence and health 27ehavior in adolescence*. Acta Paediatrica,* 100(12), 1590–1595.
15. Moksnes, U. K., Espnes, G. A., & Haugan, G. (2013). Stress, sense of coherence, and emotional symptoms in adolescents. *Psychology & Health*, 29(1), 32–49.
16. Moksnes, U. K., Espnes, G. A., & Lillefjell, M. (2012). Sense of coherence and emotional health in adolescents. *Journal of Adolescence*, 35(2), 433–441.
17. Moksnes, U. K., Rannestad, T., Byrne, D. G., & Espnes, G. A. (2011). The association between stress, sense of coherence and subjective health complaints in adolescents: Sense of coherence as a potential moderator. *Stress and Health*, 27(3), e157–e165.
18. Myrin, B. & Lagerström, M. (2008). Sense of coherence and psychosocial factors among adolescents. *Acta Pædiatrica*, 97, 805–811
19. Neuner, B., Busch, M. A., Singer, S., Moons, P., Wellmann, J., Bauer, U. (2011). Sense of coherence as a predictor of quality of life in adolescents with congenital heart defects: A register-based 1-year follow-up study. *Journal of Developmental & Behavioral Pediatrics,* 32(4), 316–327.
20. Nielsen, A. M., & Hansson, K. (2007). Associations between adolescents‘ health, stress and sense of coherence. *Stress and Health*, 23(5), 331–341.
21. Nilsson, K.W., Leppert, J., Simonsson, B., & Starrin, B. (2010). Sense of coherence and psychological well-being: improvement with age. *Journal of Epidemiological and Community Health*, 64, 347 – 352.
22. Park, Y. H., Hyun, H. J., Yu, S. J., & Byen, D. H. (2011). Factors related problem behaviors in high school girls. *Journal of Korean Academy of Community Health Nursing*, 22(3), 315-324.
23. Phi, J.H., Wang, K.C., Cho, B.K., Kim, S.K. (2008). Pediatric cerebrovascular disease. *Korean J Pediatr*. 51(12), 1282-1289.
24. Pelicand, J., Fournier, C., Le Rhun, A., & Aujoulat, I. (2015). Self-care support in paediatric patients with type 1 diabetes: Bridging the gap between patient education and health promotion? A review. Health Expectations, 18(3), 303–311.
25. Pender, N. J. (1996). Health promotion in nursing practice (3^rd^ ed.). Connecticut: *Appleton & Lange Stanford*.
26. Perminas, A, Dovile N. (2012). Changes in the Sense of Coherence of 13-17- Year-Old Adolescents after the Application of Cognitive-Behavioural Intervention. *Socialiniu Mokslu Studijos*. 4(4);
27. Sagy, S., & Braun, L. O. (2009). Adolescents under rocket fire: When are coping resources significant in reducing emotional distress? *Global Health Promotion*, 16(4), 5–15.
28. Sarah, L., Michael, J., Rivkin, A. K., Gabrielle, J. E. (2017). Moyamoya Disease in Children: Results From the International Pediatric Stroke Study. *Journal of Child Neurology*, 32(11), 924-929.
29. Selye, H. (1974). Stress without distress. *Philadelphia, PA*: J.B. Lippincott Co.
30. Scott, R. M., Smith, E. R. (2009) Moyamoya disease and moyamoya syndrome. *N Engl J Med* 360(12), 1226–1237
31. Shim, K. W., Park, E. K., Kim, J. S., & Kim, D. S.(2015), Cognitive Outcome of Pediatric Moyamoya Disease*. J Korean Neurosurg Soc.* 57 (6) , 440-444.
32. Shin, Y. M. & Cho, S. M. (2012). Emotional and behavioral problems in children with chronic physical illness. *Annals of Pediatric Endocrinology & Metabolism*, 17, 1-9.
33. Shin, Y. H., Sim, M. K., & Kim, T. I. (2006). Resilience and Health-Related Quality of Life in Children with Chronic Illness, *Journal of Korean Academy of Child Health Nursing*, 12(3), 295-303.
34. Sivertsen, B., Petrie, K. J., Wilhelmsen-Langeland, A., & Hysing, M. (2014). Mental health in adolescents with Type 1 diabetes: results from a large population-based study. *BMC Endocrine Disorders*, 14:83.
35. Smith, J., Scott, R. M. (2001). Treatment of Moyamoya syndrome in children. *Seminars in Cerebrovascular Diseases and Stroke*, 1(3), 225-239.
36. Su, S. H., Hai, J., Zhang, L., Wu, Y. F., Yu, F. (2013). Quality of life and psychological impact in adult patients with hemorrhagic moyamoya disease who received no surgical revascularization. *Journal of the Neurological Sciences*, 328(1-2), 32-36.
37. Suzuki, J., Takaku, A.(1969). Cerebrovascular “moyamoya” disease. Disease showing abnormal net-like vessels in base of brain. *Arch Neurol* , 20: 288-299.
38. .
39. Yasargil, M. G., Yonekawa, Y., Denton, I., Piroth, D., Benes, I. (1974). Experimental intracranial transplantation of autogenic omentum majus. *Journal of Neurosurgery,* ,39, 213-217.
40. Yeom, I. S., Kim, D. S., Lee, E. Y., Kim, H. S. (2015). The Characteristics of Intellectual and Psychological in the Children with Moyamoya Disease. *Child Health Nurs Res*. 21(2), 123-130.
41. Yeom, I. S., and Oh, W. O. (2018). Development and validation of a scale to measure health behavior of aldolescents suffering from moyamoya disase, *undergoing submission*.
42. Yim, S. H., Cho, C. B., Joo, W. I., Chough, C. K., Park, H. K., Lee, K. J (2002). . Prevalence and epidemiological features of Moyamoya disease in korea. *Journal of Cerebrovascular and Endovascular Neurosurgery*, 14(2):75–78.
43. Takanashi, J. I. (2011). Moyamoya disease in children. *Brain and Development*, 33(3), 229-234.
44. The National Youth Policy Institute. Korean youth indicator survey V: Health and safety (protection) [Internet]. Seoul: Author; 2010 [cited 2012 December 31]. Available from: <http://118.128.24.6/pdfs/2010/11.pdf>
45. Varni, J. W., Katz, E. R., Seid, M., Quiggins, D. J. L., and Friedman-Bender, A. (1998a). The Pediatric Cancer Quality of Life Inventory-32 (PCQL-32): I. Reliability and validity. Cancer,82: 1184–1196.
46. Varni, J. W., Seid, M., & Kurtin, P. S. (2001). PedsQL 4.0: Reliability and validity of the pediatric quality of life inventory version 4.0 genericcore scales in healthy and patient populations. *Medical Care, 39*(8), 800-812.
47. van Dyck, P. C., Kogan, M. D., McPherson, M. G., Weissman, G. R., & Newacheck, P. W. (2004). Prevalence and characteristics of children with special health care needs. Archives of *Pediatrics and Adolescent Medicine*, 158(9), 884-890.
48. Wang, Q., Hay, M., Clarke, D., & Menahem, S. (2014). Associations between knowledge of disease, depression and anxiety, social support, sense of coherence and optimism with health-related quality of life in an ambulatory sample of adolescents with heart disease. Cardiology in the Young, 24(1), 126–133.
49. Wakai, K., Tamakoshi, A., Ikezaki, K., Fukui, M., Kawamura, T., Aoki, R., Kojima, M., Lin, Y., Ohno, Y. (1997). Epidemiological features of moyamoya disease in Japan: findings from a nationwide survey. *Clin Neurol Neurosurg* , 99(S2), S1-5.
50. White, R., Walker, P., Roberts, S., Kalisky, S., & White, P. (2006). Bristol COPD Knowledge Questionnaire (BCKQ): Testing what we teach patients about COPD. *Chronic Respiratory Disease*, 3, 123-131.
51. . Ware, J. E.(1976). Scales for Measuring general health perceptions. *Health Services Research* , 11, 396- 415.
52. Zhao, M., Zhang, D., Wang, S., Zhang, Y., Wang, R., Deng, X., Gao, F., Zhao, J.(2017) Adolescents with moyamoya disease: clinical features, surgical treatment and long-term outcome. *Acta Neurochirurgica*. 159(11), 2071–2080.

**부 록 (도구 사용 허가서)**


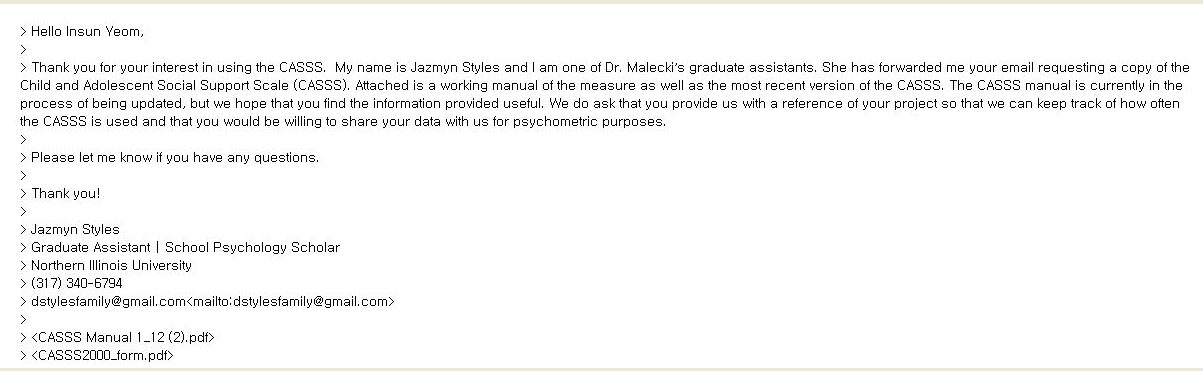


**
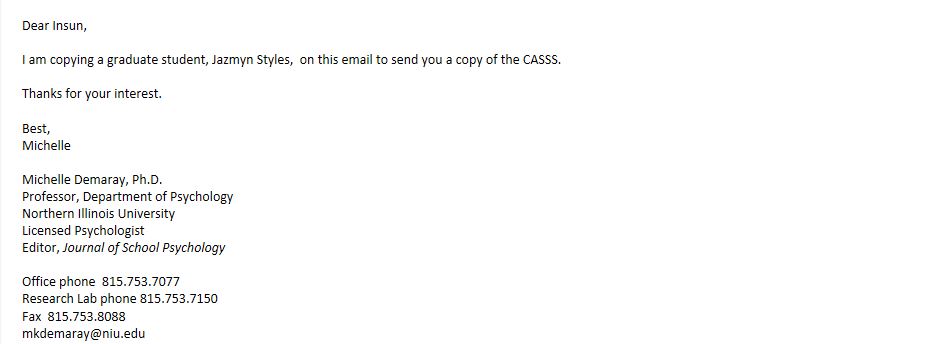
**


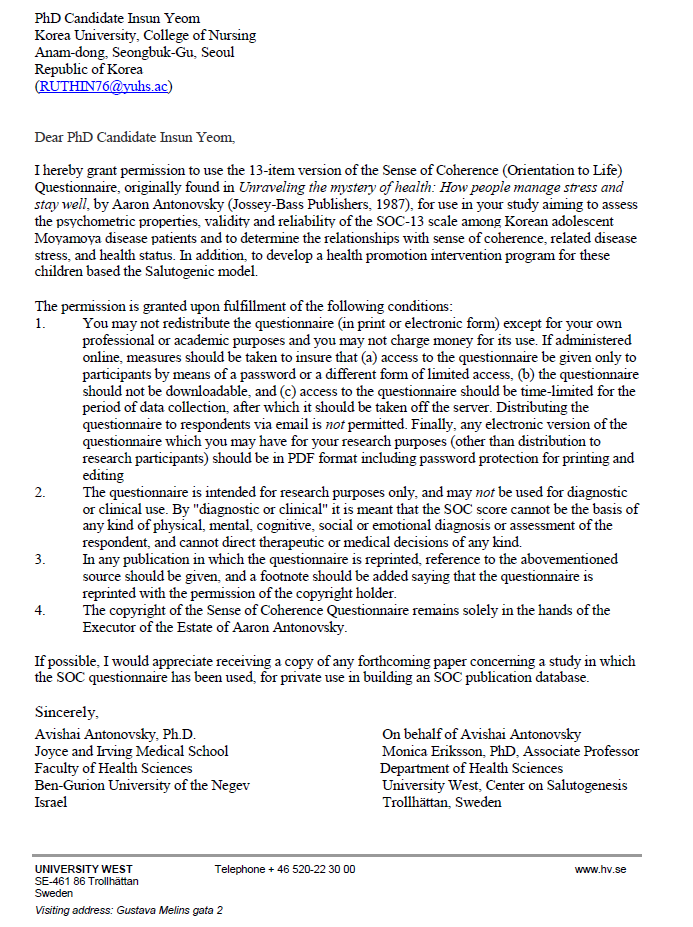

Supplement: S3 File — (DOC) [file pone.0284015.s003.doc]
